# Lis1 controls dynamics of neuronal filopodia and spines to impact synaptogenesis and social behaviour

**DOI:** 10.1002/emmm.201202106

**Published:** 2013-03-11

**Authors:** Anamaria Sudarov, Frank Gooden, Debbie Tseng, Wen-Biao Gan, Margaret Elizabeth Ross

**Affiliations:** 1 Brain and Mind Research Institute, Weill Medical College of Cornell UniversityNew York, USA; 2 Neuroscience Program, Skirball Institute, New York UniversityNew York, USA

**Keywords:** autism-relevant behaviour, cytoskeletal dynamics, Rho GTPases, schizophrenia-relevant behaviour, synapse development

## Abstract

*LIS1* (*PAFAH1B1*) mutation can impair neuronal migration, causing lissencephaly in humans. LIS1 loss is associated with dynein protein motor dysfunction, and disrupts the actin cytoskeleton through disregulated RhoGTPases. Recently, LIS1 was implicated as an important protein-network interaction node with high-risk autism spectrum disorder genes expressed in the synapse. How LIS1 might participate in this disorder has not been investigated. We examined the role of LIS1 in synaptogenesis of post-migrational neurons and social behaviour in mice. Two-photon imaging of actin-rich dendritic filopodia and spines *in vivo* showed significant reductions in elimination and turnover rates of dendritic protrusions of layer V pyramidal neurons in adolescent *Lis1*^+/−^ mice. *Lis1*^+/−^ filopodia on immature hippocampal neurons *in vitro* exhibited reduced density, length and RhoA dependent impaired dynamics compared to *Lis1*^*+/+*^. Moreover, *Lis1*^*+/−*^ adolescent mice exhibited deficits in social interaction. *Lis1* inactivation restricted to the postnatal hippocampus resulted in similar deficits in dendritic protrusion density and social interactions. Thus, LIS1 plays prominently in dendritic filopodia dynamics and spine turnover implicating reduced dendritic spine plasticity as contributing to developmental autistic-like behaviour.

## INTRODUCTION

Definition of the mechanisms regulating formation, persistence and elimination of synapses in the developing brain is a major focus in mental health research. Current hypotheses regarding neurological and neurodevelopmental disorders, such as schizophrenia, autism and various forms of mental retardation, include not only formation of faulty synaptic contacts, but also the failure to successfully prune synapses once formed and the inability to make new connections during childhood development. Excitatory synapses are located on dendritic spines, which are actin-rich protrusions located on dendritic shafts. While a majority of dendritic spines are maintained throughout life, many spines are eliminated and new spines are formed that could reflect memories lost or, through new contacts, gained (Grutzendler et al, 2002; Trachtenberg et al, 2002). During development, dendrites are adorned with long, actin-based motile filopodia that are the predecessor of dendritic spines. Dendritic filopodia rely on their motility in order to sample, test and finally make synapses to form proper circuits (Ziv & Smith, 1996). Compared to spines, relatively little is known about the regulation of filopodia dynamics and their consequence on spinogenesis, synapse formation and behaviour.

The *LIS1* gene figures prominently in neuronal motility. Total LIS1 loss causes embryonic lethality, but deletion or point mutation inactivating one *LIS1* allele causes lissencephaly, an autosomal-dominant disorder of neuronal migration. The cerebral cortical grey matter of *LIS1*^*+/−*^ patients may be thick, with a smooth surface and enlarged or absent gyri (Lo Nigro et al, 1997). In contrast, in *Lis1*^*+/−*^ mice neuronal migration lags but catches up so that brain cell patterned organization is little altered, while more subtle neuronal morphogenetic changes can occur with as little as 25% reduction in Lis1 levels (Hirotsune et al, 1998). Thus, Lis1 deficits are graded and highly gene-dose dependent. Neurologically, *Lis1*^*+/−*^ mice are fully viable, breed and have no motor impairment (Paylor et al, 1999). Clinical manifestations range from extreme mental retardation, severe epilepsy and short life span to milder forms with occasional seizures and intellectual disability (Saillour et al, 2009). A subset of patients diagnosed with *LIS1* heterozygous mutations exhibit autistic features (Saillour et al, 2009). Interestingly, LIS1 has been identified as one of the hub proteins in the functional interaction network of high-risk autism spectrum disorder (ASD) genes that act in the synapse (Neale et al, 2012), suggesting that relatively minor alterations in LIS1 may impact synaptic function. Nevertheless, how LIS1 might participate in such a complex neurobehavioural disorder as ASD is unclear.

Lis1 is widely expressed in postnatal and adult brain, including hippocampus and barrel cortex, and enriched in synaptosomal fractions (McKenney et al, 2010; Niethammer et al, 2000). While the role of Lis1 during neuronal proliferation and migration has been comprehensively studied (Vallee & Tsai, 2006), its role in post-migrational neurons remains largely unknown. Previous electrophysiological study of *Lis1*^*+/−*^ neurons showed over-excitation of excitatory hippocampal circuits as a consequence of increased presynaptic vesicle numbers per terminal (Greenwood et al, 2009). However, Lis1 post-synaptic actions during synapse development, affecting actin-rich dendritic protrusions and control of synaptic plasticity, have yet to be determined.

The molecular mechanisms underlying filopodial and spine dynamics are not entirely understood. However, Rho-family GTPases can alter dendritic spine development through regulation of the filamentous, F-actin, cytoskeleton (Nakayama et al, 2000; Tashiro et al, 2000). RhoA inactivation increases dendritic spine density and neck length, while Rac1 inhibition results in spine loss (Luo et al, 1996; Nakayama et al, 2000; Tashiro et al, 2000). RhoA activation inhibits spine formation, blocking spine head growth and stability, while Rac1 activation leads to a greater number and more stabilized dendritic spines (Nakayama et al, 2000; Tashiro et al, 2000). We previously showed that *Lis1* haploinsufficiency is associated with disregulation of Rho-family GTPases, such that RhoA activity is increased and Cdc42 and Rac1 activities are suppressed in brain tissue and neurons in primary culture (Kholmanskikh et al, 2003). Moreover, *Lis1* deficient neurons display reduced F-actin content in processes and fewer and shorter filopodia (Kholmanskikh et al, 2003, 2006). While the importance of Rho GTPases in spine formation is recognized, how Rho-family GTPase activities are modulated to regulate actin cytoskeletal rearrangements and synaptic plasticity is incompletely understood. Here, we use *in vitro* and systems-level *in vivo* approaches to interrogate the role of Lis1 in dendritic protrusion dynamics of excitatory neurons, and examine its effect on synaptogenesis and behaviour. These studies provide new insight into the molecular genetic program linking aberrant actin-based dynamics and systems level brain development.

## RESULTS

### Transcranial two-photon imaging reveals deficits in filopodia dynamics in adolescent *Lis1*^*+/−*^ animals

**Figure 1:**
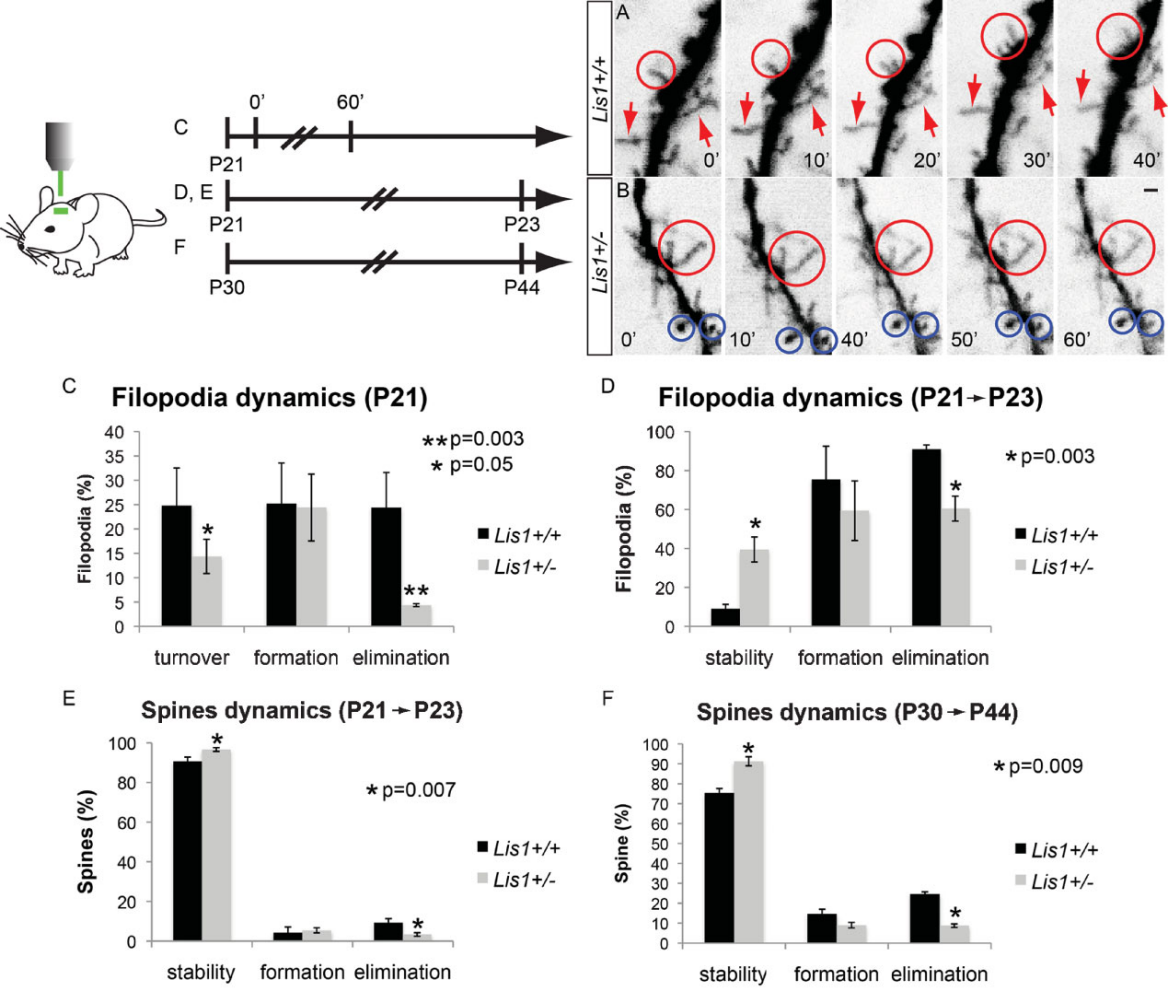
*In vivo* two photon time lapse imaging in adolescent and young adult *Lis1^+/+^* and *Lis1^+/−^* mice. A. *Lis1*^*+/+*^ neurons have multiple filopodia that are very dynamic (encircled). Arrows indicate other stable filopodia. Images are color inverted. (*n*_+/+_ = 4 animals, *n*_+/−_ = 3 animals; *n*_+/+_ = 269 total spines, *n*_+/−_ = 205 total spines). Scale bar: 2 µm. B. *Lis1*^*+/−*^ neurons have fewer dynamic filopodia (*e.g.* filopodia that neither retract nor extend during imaging period are encircled in red, blue circles depict spines for comparison). C–F. Quantification of filopodia and spine dynamics in adolescent (P21) and young adult (P30) *Lis1*^*+/+*^ and *Lis1*^*+/−*^ mice. C. Filopodia elimination (*p* = 0.003) and turnover (*p* = 0.05) rates are lower in *Lis1*^*+/−*^ mice over 1 h (1st view at 0′ followed by 2nd view at 60′), whereas formation rates were similar (*p* = 0.9). D. Filopodia stability (*p* = 0.003) increased over 2 days (1st view at P21, 2nd view of the same region at P23) in adolescent *Lis1*^*+/−*^ mice compared to control mice. In contrast, the elimination (*p* = 0.003) rate in *Lis1*^*+/−*^ mice decreased over 2 days relative to controls. Formation rates trended lower but did not reach significance (*p* = 0.2). (*n*_+/+_ = 4 animals, *n*_+/−_ = 3 animals; *n*_+/+_ = 182 total filopodia, *n*_+/−_ = 88 total filopodia in C; *n*_+/+_ = 173 total filopodia, *n*_+/−_ = 135 total filopodia in D). E. Quantification of spine dynamics in young adult mice over 48 h (1st view at P21, 2nd view of the same region at P23) reveals increased stability (*p* = 0.007), decreased elimination (*p* = 0.007) and no difference in formation rates. (*n*_+/+_ = 4 animals, *n*_+/−_ = 3 animals; *n*_+/+_ = 246 total spines, *n*_+/−_ = 220 total spines). F. Quantification of spine dynamics in adult mice over a 2-week period (1st view at P30, 2nd view of the same region at P44) show increased stability (*p* = 0.009) and decreased elimination (*p* = 0.009) rates in *Lis1*^*+/−*^ mutants, while again, formation showed a reduced trend (*p* = 0.43).

Dendritic filopodia make initial synaptic contacts while actively exploring their environment (Dailey & Smith, 1996; Ziv & Smith, 1996). We compared filopodia dynamics in *Lis1*^*+/+*^ and *Lis1*^*+/−*^ fluorescently labelled layer V pyramidal neurons, whose projections extend to superficial lamina in the mouse barrel cortex, using transcranial two-photon microscopy (Grutzendler et al, 2002; Yang et al, 2010). Dendritic segments in 3-week-old adolescent animals were imaged every 10 min over a 1 h period, or over a 2 day interval. Significant decreases occurred in turnover and elimination rates of filopodia in *Lis1*^*+/−*^ animals over 10 min (Fig 1A and B, Supporting Information Fig 1A). Consistent with previous studies showing high turnover rates of dendritic filopodia (Dailey & Smith, 1996; Dunaevsky et al, 1999; Grutzendler et al, 2002; Lendvai et al, 2000; Ziv & Smith, 1996), 24.4 ± 7% of filopodia were eliminated, 25.2 ± 8% formed, with an overall turnover rate of 24.8 ± 7% in *Lis1*^*+/+*^ animals over 1 h (Fig 1A and C). In contrast, rates in *Lis1*^*+/−*^ animals were significantly decreased to only 4.4 ± 0.3% of filopodia eliminated and 14.4 ± 3% of filopodia turned over in this interval, whereas formation rates were similar (24.4 ± 7%; Fig 1B and C). Dendritic segments were also imaged *in vivo* at P21 and again at P23. In controls, most filopodia present at P21 were eliminated by P23 (90.9 ± 2.7%), many were formed (75.4 ± 17%) and very few remained (∼9.1 ± 2%; Fig. 1D). Filopodia dynamics in *Lis1*^*+/−*^ animals were starkly different, eliminating fewer filopodia over two days (60.5 ± 6.4% in *Lis1*^*+/−*^), leaving more intact (39.5 ± 6.4% in *Lis1*^*+/−*^) and forming somewhat fewer protrusions compared to wildtype (59.4 ± 8.15% in *Lis1*^*+/−*^; Fig. 1). Furthermore, we found overall decreased density in the number of filopodia present in *Lis1* happloinsufficient barrel cortex at P21 in comparison to control animals (Supporting Information Fig 1B). Thus, two-photon time-lapse imaging suggests that *Lis1* has a crucial role in filopodia dynamics in adolescent intact brain.

### Increased stability and decreased elimination rates of dendritic spines in *Lis1*^*+/−*^ mice

Previous studies have shown that dendritic spines are mostly stable over hours but undergo turnover over days and weeks (3,4). We next asked whether *Lis1* haploinsufficiency affects spine dynamics as well, using two-photon microscopy in adolescents over 2 days and in young adult animals by repeated imaging of the same dendrites at P30 and P44. Consistent with filopodia results, we recorded in adolescent *Lis1*^*+/−*^ mutants a significant decrease in spine elimination over 48 h (*Lis1*^*+/+*^: 9.3 ± 2%, *Lis1*^*+/−*^: 3.3 ± 0.9%), a significant increase in stability (*Lis1*^*+/+*^: 90.7 ± 2%, *Lis1*^*+/−*^: 96.7 ± 0.9%) and no difference in formation rates (*Lis1*^*+/+*^: 4.3 ± 3%, *Lis1*^*+/−*^: 5.4 ± 1.3%; Fig. 1E). Similarly, in young adults over a 2-week period, we found a significant increase in spine stability (*Lis1*^*+/+*^: 75.4 ± 5%, *Lis1*^*+/−*^: 91.3 ± 1.6%), and a significant decrease in the rate of spine elimination (*Lis1*^*+/+*^: 24.6 ± 5%, *Lis1*^*+/−*^: 8.7 ± 1.6%) in *Lis1*^*+/−*^ animals (Fig 1F, Supporting Information Fig 1C and D). Spine formation rates tended to be lower in the *Lis1* mutants but differences did not reach statistical significance between genotypes (*Lis1*^*+/+*^: 14.6 ± 10%, *Lis1*^*+/−*^: 8.9 ± 4%). However, we found no differences in the filopodia to spine ratio between *Lis1*^*+/+*^ and *Lis1*^*+/−*^ animals (Supporting Information Fig 1E and F). Therefore, reduction in filopodia density is accompanied by a proportional reduction in spine density. These data indicate that Lis1 is required for promoting both filopodia and spine dynamics, with a crucial role in pruning (elimination) of dendritic protrusions.

### Lis1 reduction impairs filopodia density and motility

**Figure 2:**
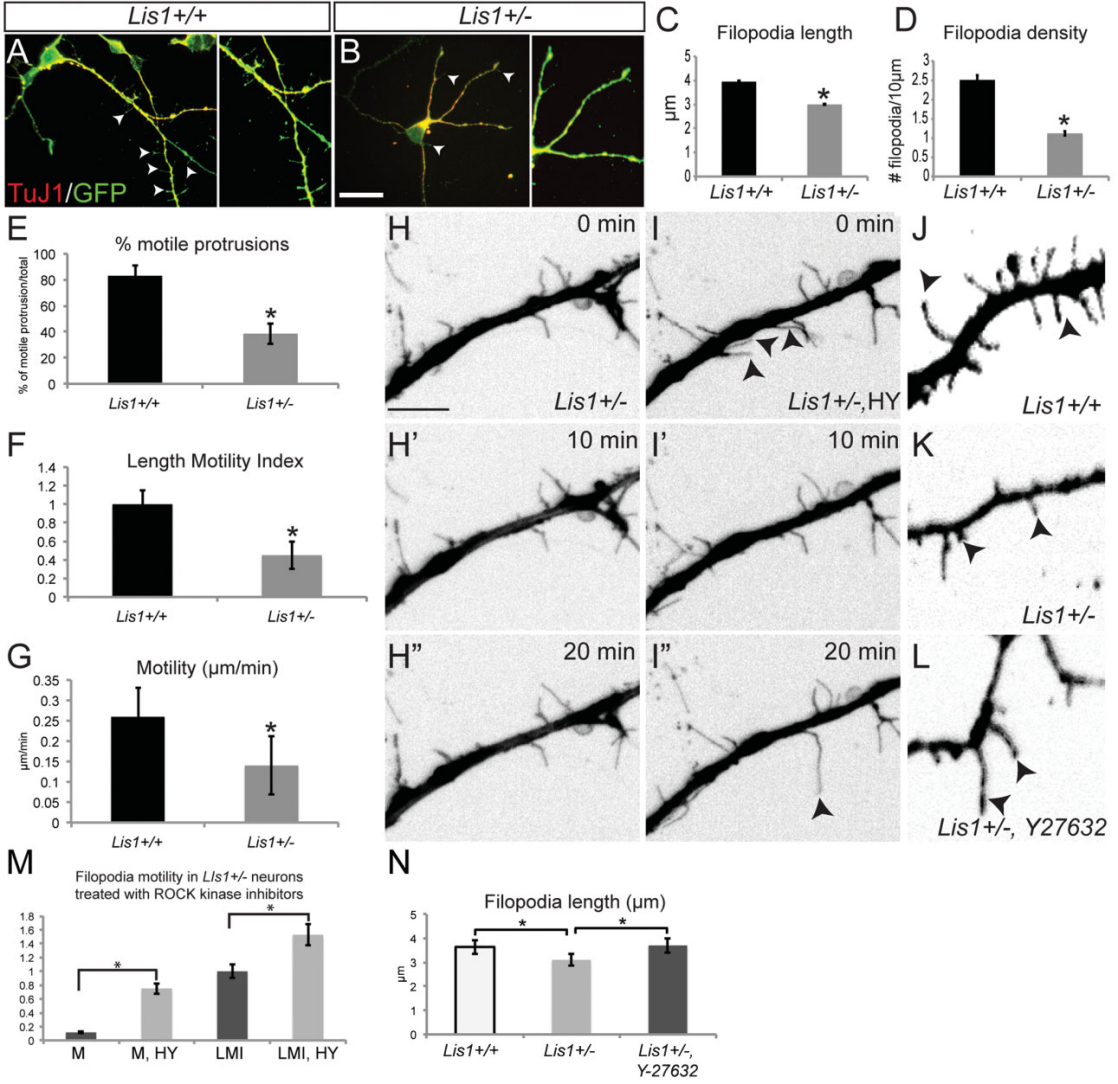
Downregulation of RhoA in *Lis1^+/−^* neurons rescues motility in a subset of filopodia. A,B. *Lis1*^*+/+*^ (A) and *Lis1*^*+/−*^ (B) DIV2 hippocampal neurons stained with TuJ1 (red) and GFP for myr-Venus. Scale bar 10 µm. C. Filopodial length quantification shows shorter protrusions in *Lis1*^*+/−*^ neurons at DIV2 (*p* = 0.01). D. Filopodial density is reduced 60% in *Lis1*^*+/−*^ neurons at DIV2 (*p* = 0.001). (*n*_+/+_ = 10 neurons, *n*_+/−_ = 7 neurons; 10–15 filopodia/neuron). E. Number of motile filopodia at DIV2 presented as a percentage of total filopodia per cell (*p* = 0.001). F,G. Length Motility Index (F) and Motility (G) were significantly less in *Lis1*^*+/−*^ neurons (*p* = 0.001). (*n*_+/+_ = 10 neurons, *n*_+/−_ = 7 neurons; 10–15 filopodia/neuron). H–H''. Time lapse video of a DIV2 *myrVen;Lis1*^*+/−*^ hippocampal neuron. Note absence of dynamic filopodia in this part of dendrite. I–I''. Time lapse video of the same dendrite after 1 h incubation with ROCK inhibitor cocktail. Filopodia marked with arrowheads extend and retract. Scale bar 5 µm (H–I″). J–L. Filopodia length increases in *Lis1*^*+/−*^*;myrVen* neurons after 4 h incubation with 10 µM Y27632. H–L images are color inverted. Scale bar 2 µm (J–L). M. Quantification of M (motility) and LMI (length motility index) shows increased rates in filopodia of *Lis1*^*+/−*^ neurons treated with ROCK inhibitors, 10 µM HA1077 + 10 µM Y27632 (HY) (*n*_total filopodia_ = 26; *p* = 0.0005). 25–30% of non-motile filopodia in *Lis1*^*+/−*^*;myrVen* neurons exhibited rescued motility post drug treatment. N. Filopodia length is significantly increased in *Lis1*^*+/−*^*;myrVen* neurons after 4 h incubation with 10 µM Y27632 (*n* = 250 filopodia/genotype; *p* = 0.01).

To further probe the functional role of Lis1, we examined immature, living cultured hippocampal neurons using spinning-disc confocal microscopy. *Lis1*^*+/−*^ animals were mated with a *CAG::myr-Venus* strain that widely expresses a myristoyl-Venus yellow fluorescent fusion protein, suited for the visualization of neuronal filopodia (Rhee et al, 2006). Indeed, *Lis1*^*+/−*^;*myr-Ven* immature neurons cultured for only 2 days *in vitro* (DIV2) had fewer and shorter filopodia compared to control neurons (Fig 2A–D). Time-lapse recordings showed significantly reduced motile filopodia numbers in *Lis1*^*+/−*^ neurons (83 ± 10.3% of *Lis1*^*+/+*^ *vs.* 38.6 ± 3.5% of *Lis1*^*+/−*^, Fig 2E, Supporting Information Video 1). In control neurons, many filopodia extended, retracted, bent and/or disappeared and appeared throughout the time-lapse video (Supporting Information Video 1), whereas mutant neuronal filopodia underwent very few extensions or retractions. To quantify motility, filopodia tips were tracked with a red mark at each time point (Supporting Information Fig 2). In *Lis1*^*+/+*^*;myr-Ven* neurons, marks were dispersed, while in *Lis1*^*+/−*^*;myr-Ven* neurons many were superimposed, indicating absence of movement (Supporting Information Fig 2). Filopodium extension and retraction rates were quantified using a ‘Length Motility Index’ (LMI; Brocco et al, 2010; Konur & Yuste, 2004). *Lis1*^*+/−*^ neuron LMIs were lower than controls (*Lis1*^*+/+*^: 1.00 ± 0.3; *Lis1*^*+/−*^: 0.45 ± 0.25; Fig 2F). Filopodial movement velocities of *Lis1*^*+/−*^*;myr-Ven versus Lis1*^*+/+*^*;myr-Ven* were significantly diminished (*Lis1*^*+/+*^: 0.25 ± 0.1 µm/min; *Lis1*^*+/−*^: 0.14 ± 0.06 µm/min; Fig 2G). Therefore, not only does Lis1 influence the density of filopodia in immature neurons, but also filopodial length and motility, similar to deficits observed in barrel cortex *in vivo*.

### RhoA inhibition restores dynamics of a filopodial subset in *Lis1*^*+/−*^ neurons

Since *Lis1* haploinsufficiency leads to elevated RhoA activity (Kholmanskikh et al, 2003, 2006) and constitutive activation of RhoA can impact spine formation and stability (Tashiro et al, 2000), we asked whether upregulated RhoA is responsible for diminished filopodial motility in *Lis1*^*+/−*^ neurons. As Rho kinase (ROCK) is the major downstream effector of RhoA (Fukata et al, 2001), we used a cocktail of two specific ROCK inhibitors (Uehata et al, 1997), 10 µm Y-27632 and 10 µm HA1077, to effectively reduce RhoA activity (Kholmanskikh et al, 2003). Taking advantage of the ease of pharmacological manipulations *in vit*ro, time-lapse images of dendritic filopodia were followed by a bath application of the cocktail for 1 h before a repeated time-lapse recording of the same filopodia. Consistent with previous studies (Govek et al, 2005; Kholmanskikh et al, 2003), no differences in filopodia motility or length occurred upon application of ROCK inhibitors on *Lis1*^*+/+*^ neurons (*Lis1*^*+/+*^ 3.64 ± 0.8 µm; *Lis1*^*+/+*^HY 3.61 ± 1 µm). In contrast, in the presence of ROCK inhibitor, 25–30% of non-motile *Lis1*^*+/−*^ filopodia displayed significantly increased motility, LMI and length (filopodia, *n* = 26; Fig. 2H–I, M, J–L, N; Supporting Information Video 2). We conclude that elevated RhoA activity in *Lis1*^*+/−*^ neurons significantly contributes to their decreased filopodia dynamics.

### Actin polymerization defects in dendritic protrusions of *Lis1*^*+/−*^ neurons

**Figure 3:**
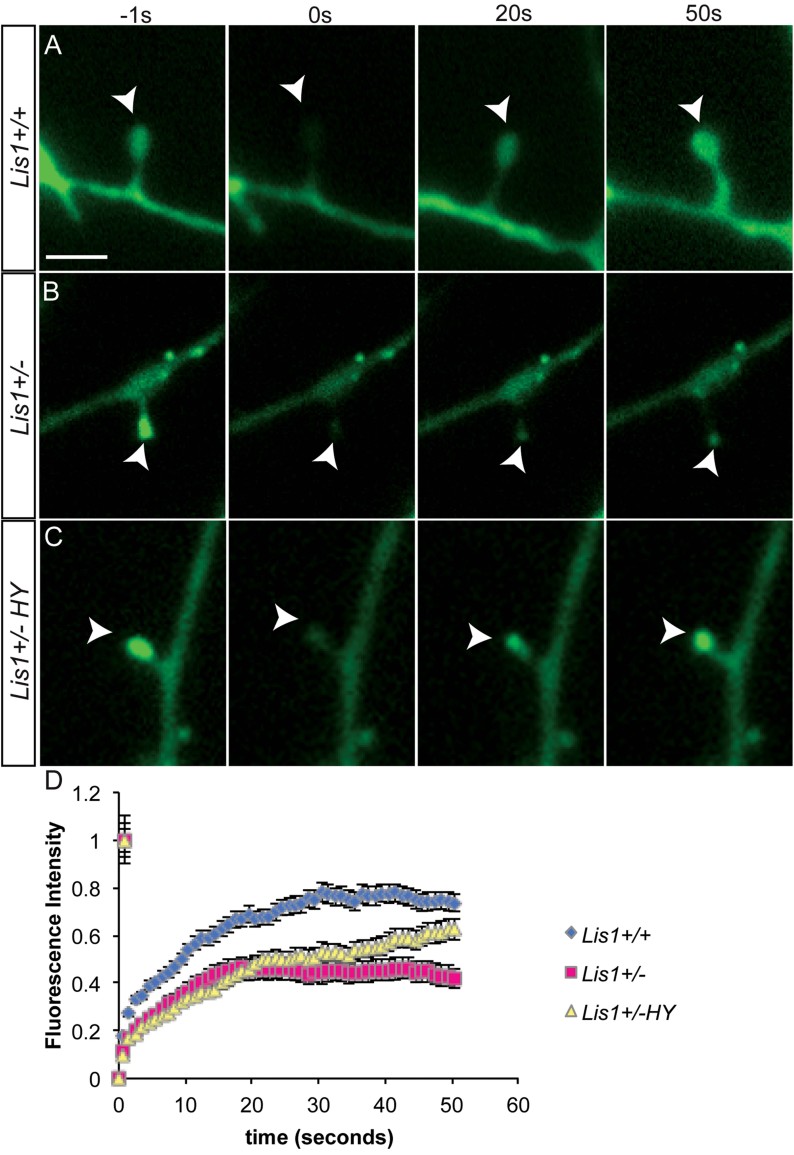
Rho kinase inhibitors can rescue Actin polymerization defects in Lis1^+/−^ neurons. A–C. Incorporation of fluorescently labelled actin monomers into filamentous actin in spines of mouse hippocampal neurons was visualized using FRAP at DIV12–14. GFP-actin was photobleached from the spines and time-lapse imaging followed the fluorescence recovery of F-actin. *Lis1*^*+/−*^ neurons were treated with 10 µM HA1077 + 10 µM Y27632 (HY) for 1 h before imaging (*Lis1*^*+/−*^ HY). Scale bar 4 µm. D. The averaged recovery curves from *Lis1*^*+/+*^ (blue), *Lis1*^*+/−*^ (pink) and *Lis1*^*+/−*^ HY (yellow) revealed a diminished recovery in *Lis1*^*+/−*^ neurons, but significant rescue in *Lis1*^*+/−*^ HY. (*n*_+/+_ = 35 total protrusions, *n*_+/−_ = 25 total protrusions, *n*_+/−HY_ = 18 total protrusions).

Actin polymerization, depolymerization, capping and severing of F-actin largely determine dendritic protrusion dynamics. To test whether Lis1 affects actin dynamics, dendritic filopodia and spine actin incorporation into filaments was compared in *Lis1*^*+/+*^ and *Lis1*^*+/−*^ dissociated hippocampal neurons. Cultures were infected with AAV2.GFP-Actin on DIV0 and examined using fluorescence recovery after photobleaching (FRAP) at DIV12-14. GFP-Actin fluorescence was recovered to 73.6 ± 3.9% in wild type neurons in 50 s (Fig 3A and D; Supporting Information Video 3A). In contrast, recovery in *Lis1* haploinsufficient neurons was significantly slower (*p* = 0.0001), achieving only 41.8 ± 3.1% in 50 s (Fig 3B and D; Supporting Information Video 3B). To test whether reducing RhoA activity in *Lis1*^*+/−*^ neurons leads to faster recovery of GFP-actin fluorescence, mutant neurons were treated with the ROCK inhibitors for 1 h prior to FRAP. The *Lis1*^*+/−*^ phenotype was substantially rescued in the presence of ROCK inhibitors, where GFP-Actin fluorescence was significantly recovered (*p* = 0.03) to 62.5 ± 1.9% in 50 s (Fig 3C and D; Supporting Information Video 3C). Visualization of fluorescently labelled actin monomers indicated that, compared to wild type, actin polymerization is greatly diminished in *Lis1*^*+/−*^ neurons, which could explain the observed reduction in dendritic protrusion dynamics *in vitro* and *in vivo*.

### *Lis1* deficit during development results in simplified dendritic structure and reduced spine density in CA1 pyramidal neurons in adolescent animals

**Figure 4:**
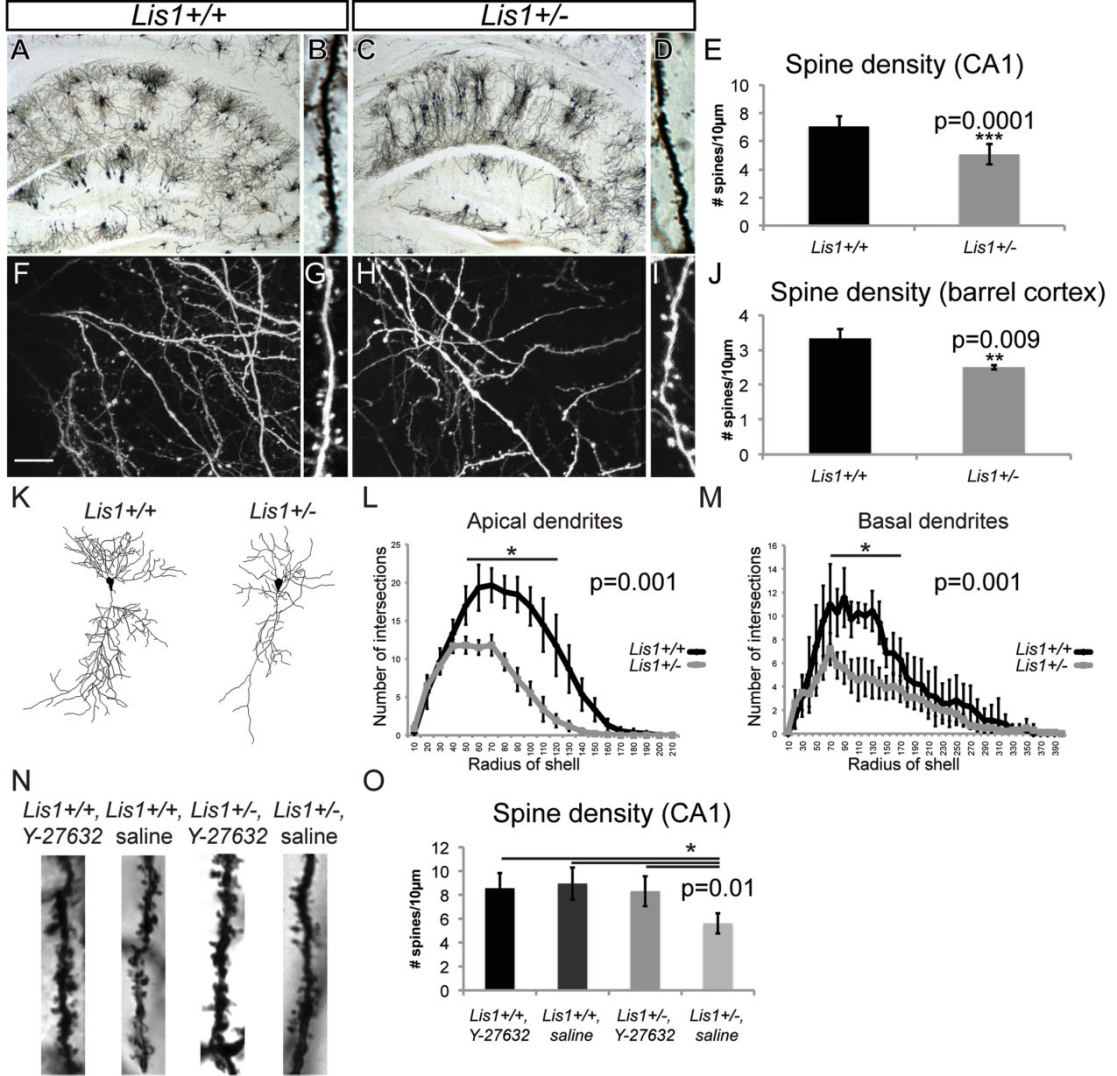
Reduced spine density in young pyramidal neurons of *Lis1^+/−^* mice. A–D. Golgi-stained hippocampal CA1 pyramidal neurons in brain sections of control (A, B) and *Lis1*^*+/−*^ (C, D) P21 litter-mates. Scale bar 50 µm (A, C), 10 µm (B, D). E. Quantification of spine density along dendrites of hippocampal CA1 pyramidal neurons in *Lis1*^*+/+*^ and *Lis1*^*+/−*^ mice (*n* = 110 dendritic segments per genotype; *p* = 0.0001). F–I. Two-dimensional projection of a three-dimensional stack of dendritic branches in *Lis1*^*+/+*^ (F,G) and *Lis1*^*+/−*^ (H,I) barrel cortex. Scale bar 15 µm (F,G). J. Spine density quantification in barrel cortex (*n* = 3/genotype; *p* = 0.009). K. Neurolucida drawings of pyramidal neurons representative for both genotypes. L,M. Quantification of the number of intersections per circle. (L) Significant differences were found in the circles ranging from 50 to 120 µm of apical dendrites in *Lis1*^*+/+*^ compared to *Lis1*^*+/−*^ (*n* = 10 neurons/genotype; *p* = 0.001). (M) Significant differences were found in the circles ranging from 60 to 160 µm of basal dendrites in *Lis1*^*+/+*^ compared *to Lis1*^*+/−*^ (*p* = 0.001). N. Golgi-stained hippocampal CA1 pyramidal neurons in sections from *Lis1*^*+/+*^ and *Lis1*^*+/−*^ mice collected 24 h after i.p. injection of saline or Y-27632 (*n* = 3/genotype/treatment; 60 dendritic segments/animal). Scale bar 10 µm. O. Quantification of spine density along apical CA1 pyramidal dendrites in *Lis1*^*+/+*^ and *Lis1*^*+/−*^ mice treated with saline or Y-27632 for 24 h (*p* = 0.01).

The heterozygous deletion of *Lis1* is associated with distinct lamination abnormalities of the hippocampal CA1 region but no difference in spine density was observed in *Lis1*^*+/−*^ adult mice older than 2–3 months (Fleck et al, 2000). Furthermore, we found no difference in the spine density of mature dissociated hippocampal neurons cultured for 21 days *in vitro* (Supporting Information Fig 3). Because we observed significant alterations in filopodia and spine dynamics in young mutant neurons, we asked whether *Lis1* haploinsufficiency would affect the geometry of dendritic branches and spine density in adolescent mice. Hippocampi at P21 were visualized using Golgi staining. Compared to controls, the overall organization of dendritic branches of individual CA1 neurons appeared altered in *Lis1*^*+/−*^ animals (Fig 4A and C). Dendrites of CA1 pyramidal neurons in these young mutant animals were more columnar, while secondary and tertiary branches remained close to primary dendrites (Fig 4C). Despite the abnormal shape of the *Lis1*^*+/−*^ pyramidal neuron dendritic tree, cells appeared healthy without any signs of degeneration. More detailed analysis revealed a striking decrease in spine density of CA1 pyramidal neuron apical dendrites (Fig 4B and D). Linear spine density in mutant neurons was reduced to 70% of control (*Lis1*^*+/+*^: 7.04 ± 0.5 spines/10 µm; *Lis1*^*+/−*^: 5.05 ± 0.3 spines/10 µm; Fig 4E). Spine density was also examined in the barrel cortex of young adult (P30) *Lis1*^*+/+*^;*Thy1-YFP* and *Lis1*^*+/−*^;*Thy1-YFP* animals. Like hippocampal pyramidal neurons, apical dendrites of layer V neurons in mutants had a ∼30% lower spine density (Fig 4F–H). Sholl analysis of CA1 pyramidal neurons stained with the Golgi technique allowed the quantification of branching (Fig 4K–M). Regarding apical dendrites *Lis1*^*+/−*^ animals had significantly fewer dendritic intersections than *Lis1*^*+/+*^ animals at the proximal 50 µm to 120 µm region (*p* = 0.001; Fig 4L). Similarly, basal dendrites were affected in *Lis1*^*+/−*^ animals, so that in the region from 60 to 160 µm from the cell body there were significantly fewer intersections in comparison to *Lis1*^*+/+*^ (*p* = 0.001; Fig 4M). To test whether inhibition of hyperactive RhoA would lead to anatomical changes in adolescent *Lis1*^*+/−*^ animals we intraperitoneally injected 10 mg/kg of ROCK inhibitor Y-27632 (Fig 4N and O). As expected from previous studies, Y-27632 did not change spine density in wild type CA1 pyramidal neurons (Nakayama et al, 2000). However, ROCK inhibition in *Lis1* happloinsufficient animals restored spine densities to control levels (Fig 4O). Thus, Lis1 haploinsufficiency is associated with reduced dendritic structure and spine density in both the hippocampus and the barrel cortex of adolescent and young adult animals due to RhoA disregulation.

### Synaptic cluster formation is delayed in *Lis1*^*+/−*^ hippocampal neurons

**Figure 5:**
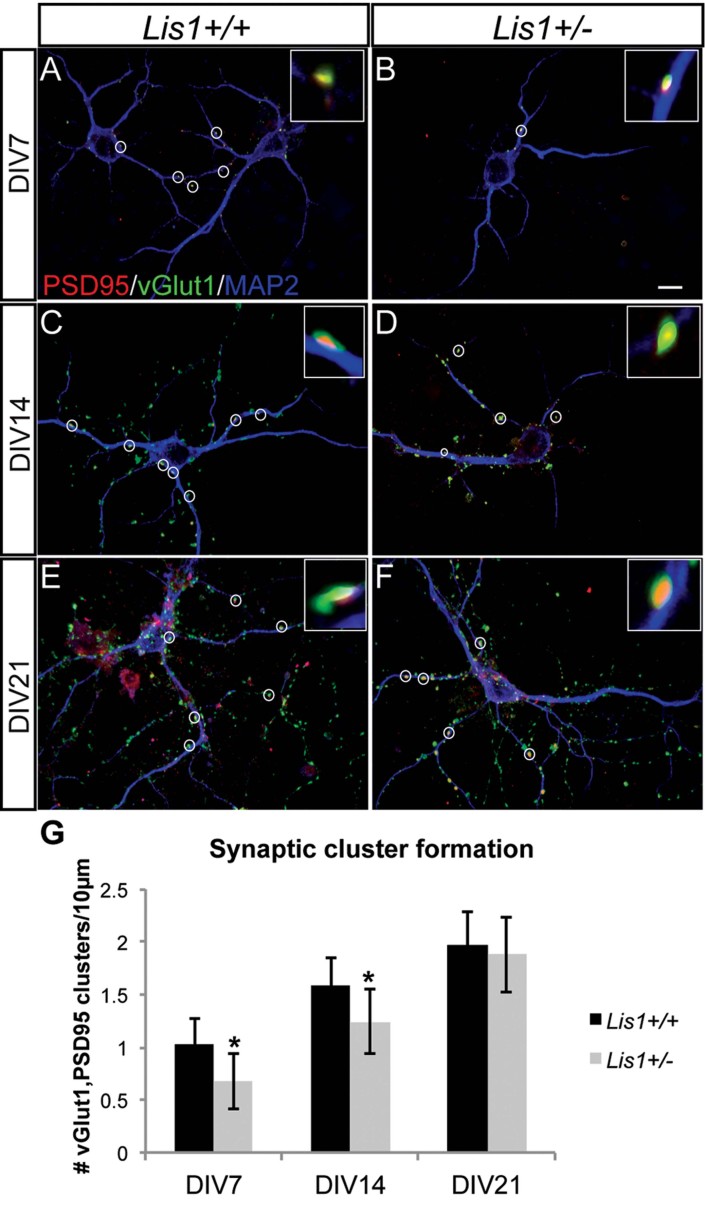
New synaptic clusters depend on Lis1 levels. A–F. Hippocampal cultures from *Lis1*^*+/+*^ (A,C,E) & *Lis1*^*+/−*^ (B,D,F), analysed at DIV7 (A,B), DIV14 (C,D) and DIV21 (E,F) after immunostaining of pre- (vGlut1) & post- (PSD95) synaptic components. Only puncta were counted with clear overlap of the two markers (circles). Scale bar 10 µm. G. Mean synaptic cluster density of *Lis1*^*+/+*^ and *Lis1*^*+/−*^ neurons at DIV7 (*p* = 0.01), DIV14 (*p* = 0.05) & DIV21. (*n* = 3–4 animals/genotype; 5–10 neurons per animal).

Filopodia and spine dynamics are linked to synapse formation and elimination, which in turn is responsible for correct circuit development. To determine whether Lis1 regulates synapse formation *in vitro*, we quantified putative synapses in dissociated *Lis1*^*+/−*^ hippocampal neurons by staining with antibodies specific for synaptic proteins (PSD95 postsynaptic; vGlut1, presynaptic; MAP2, dendrites). Punctate staining (inserts in Fig 5) was detected along MAP2 positive dendrites, and only puncta where a PSD95/vGlut1 overlap was obvious were counted as potential synapses. In DIV7 and DIV14 neurons, synaptic cluster densities were significantly lower in *Lis1*^*+/−*^ neurons *versus* wild type (Fig 5A, B, and G). However, by DIV21, synaptic cluster densities were equivalent between wild type and *Lis1*^*+/−*^ neurons (Fig 5C–G). This indicates a significant delay in the formation of synaptic clusters, with an eventual ‘catch up’ of synapses in more mature neurons.

### Reduced social interaction and abnormal social novelty recognition in *Lis1*^*+/−*^ adolescents

**Figure 6:**
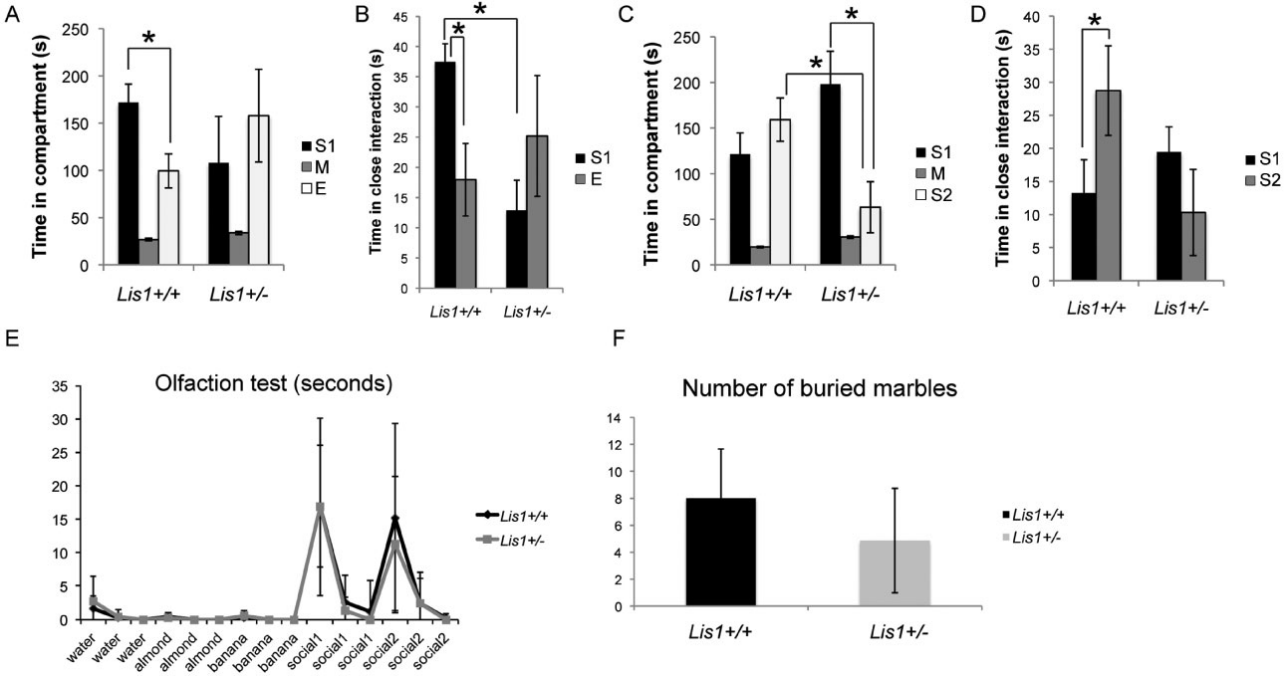
*Lis1^+/−^* mutant mice exhibit asocial behaviour in a 3-chamber social interaction test. Abbreviation: S1, stranger 1; M, medial; E, empty; S2, stranger 2. A–D. In the first phase (A,B; *p* = 0.01), the mouse is presented in one chamber with an empty cup and in another chamber with a strange mouse in the cup (stranger 1). The second phase of the test (C,D; *p* = 0.01) examines social novelty where the test mouse is presented with a novel strange mouse (stranger 2) in the previously empty cup. Total time spent in each compartment is measured (A,C), as well as time spent in close proximity/interaction with a cup/stranger mouse (B,D). *Lis1*^*+/−*^ mice spent significantly more time interacting with an empty cup (B) and failed to show interest in social novelty (D). E. As a control, *Lis1*^*+/−*^ mice performed equally well as *Lis1*^*+/+*^ mice upon testing olfaction (*n*_+/+_ = 12 animals, *n*_+/−_ = 10 animals). F. Quantification of buried marbles shows no difference between *Lis1*^*+/+*^ and *Lis1*^*+/−*^ animals.

Neurodevelopmental disorders are often associated with distinct neurobehavioural deficits in social interactions, communication, and repetitive behaviours. We hypothesized that decreased dynamics of filopodia and spines during adolescence might affect social behaviour in *Lis1*^*+/−*^ mice. Adult *Lis1*^*+/−*^ animals have spatial memory deficits on Morris water maze testing (Paylor et al, 1999), but whether adolescent animals (4 weeks old) have social behavioural deficits as well is unknown. We investigated adolescent mutant mice using a modified three-chamber social arena (Moy et al, 2004). In this test, animals can voluntarily initiate social interactions and discriminate social novelty. First, adolescent animals were allowed to freely explore the three chambers and initiate social contact with a partner (‘Stranger 1’) held in a wire cup or an identical but empty (‘Empty’) wire cup. Both the time spent in the compartment and the time spent in close interaction (sniffing, physical contact) with either ‘Stranger 1’ or an ‘Empty’ wire cup were tracked. *Lis1*^*+/−*^ animals displayed abnormal social behaviour, spending more time in the compartment and in close proximity with an empty cup as opposed to ‘Stranger 1’ (Fig 6A and B). During social novelty recognition testing, a novel ‘Stranger 2’ was introduced into the previously empty cup. Adolescent *Lis1*^*+/+*^ mice showed a preference for the compartment and close interaction with ‘Stranger 2’. In contrast, *Lis1*^*+/−*^ mice showed no preference for close interactions with either ‘Stranger 1’ or ‘Stranger 2’ (Fig 6C and D). Olfaction cues have an instrumental role in mouse social behaviours. The possibility that social behaviour deficits could result from olfaction deficits was assessed by comparing *Lis1*^*+/+*^ and *Lis1*^*+/−*^ animals in an olfactory habituation/dishabituation test (Yang & Crawley, 2009). Both genotypes showed similar abilities across three non-social and two social odours (Fig 6E). Additionally, we assessed repetitive digging behaviour, using the marble burying test as a measure of repetitive and preservative behaviour (Thomas et al, 2009). In the test cage, mice were scored after 30 min by counting the number of marbles that were more than 50% buried (Silverman et al, 2010). We found no differences between *Lis1*^*+/+*^ and *Lis1*^*+/−*^ young adult animals (Fig 6F).

### Conditional deletion of Lis1 in the adolescent hippocampus

**Figure 7:**
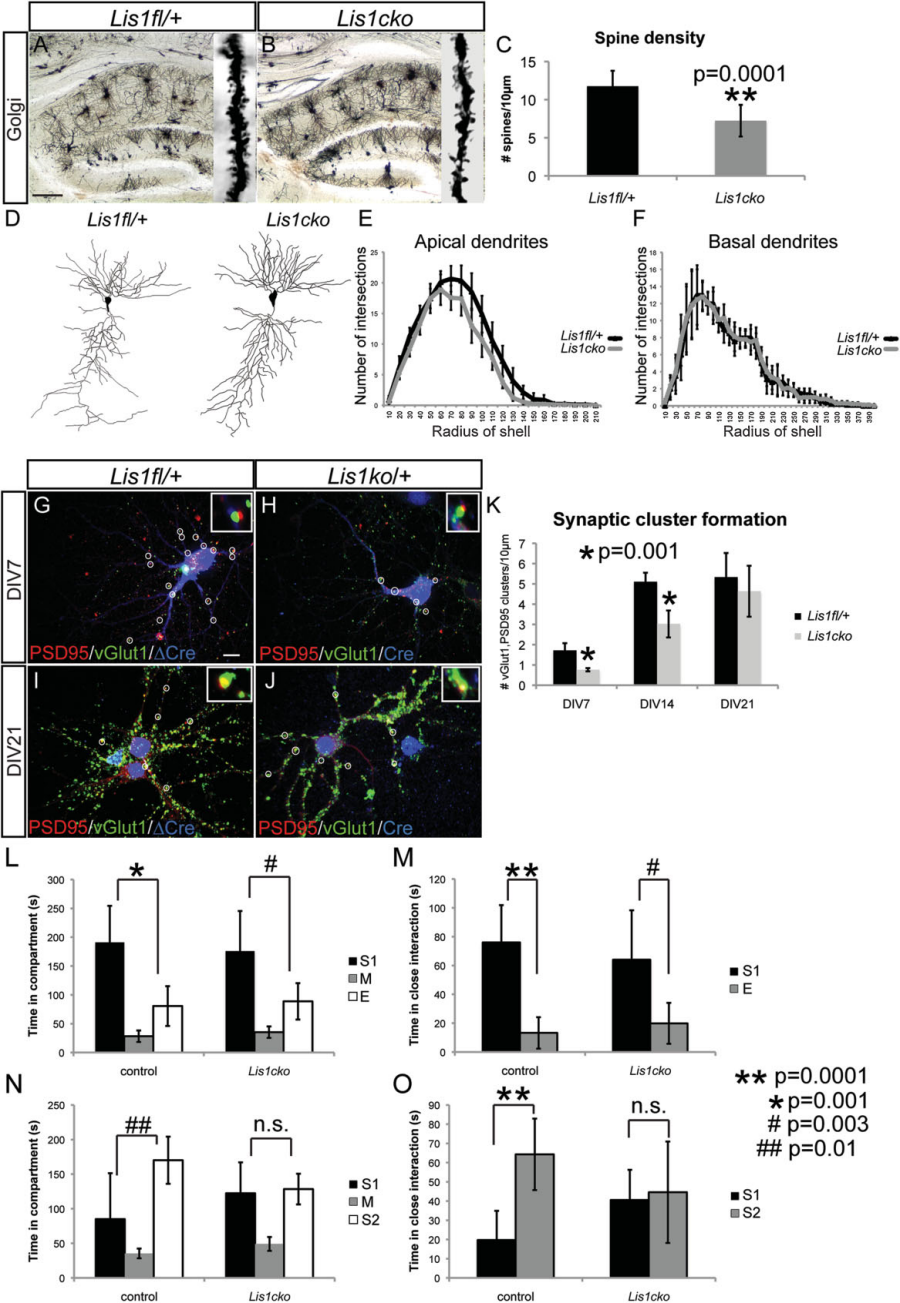
*Lis1cko* mutant mice exhibit deficits in spine density, synaptic cluster formation and social novelty recognition. A,B. Images of Golgi-stained hippocampal CA1 pyramidal neurons in *Lis1*^*flox/+*^ (A) and *Lis1cko* (B) P28 litter-mates. C. Quantification of spine density (*n* = 70 dendritic segments per genotype; *p* = 0.0001; scale bar 50 µm). D. Neurolucida drawings of pyramidal neurons representative for *Lis1*^*fl/+*^ and *Lis1cko*. E,F. Quantification of the number of intersections per circle revealed no differences in apical or basal dendrites in *Lis1*^*fl/+*^ compared to *Lis1cko* animals (P28; *n* = 10 neurons/genotype). G–J. Hippocampal cultures from *Lis1*^*flox/+*^ (infected with inactive, ▵Cre lentivirus) (G,I) and *Lis1*^*cko/+*^ (*Lis1*^*flox/+*^ neurons infected with Cre lentivirus) (H,J), analysed at DIV7 (G,H), and DIV21 (I,J) after immunostaining of pre- (vGlut1) & post- (PSD95) synaptic components. *Lis1*^*flox/+*^ cultures are infected on DIV1 with either EGFP-Cre or EGFP-inactiveCre lentivirus. GFP is used to detect Cre in infected neurons. K. Mean synaptic cluster density of *Lis1*^*flox/+*^ and *Lis1*^*cko/+*^neurons at DIV7 (*p* = 0.001), DIV14 (*p* = 0.001) & DIV21. (*n* = 3–4 animals/genotype; 5–10 neurons per animal; scale bar 10 µm). L–O. *Lis1cko* mutant mice exhibit deficits in social behaviour testing. Both control and *Lis1cko* mice show preference for a stranger mouse in the cup (S1) as opposed to an empty cup (E). In contrast to controls, *Lis1cko* mice show no preference for social novelty (stranger 2) in the previously empty cup (*n*_fl/+_ = 11 animals, *n*_cko_ = 10 animals). Abbreviation: S1, stranger 1; M, medial; E, empty; S2, stranger 2.

In order to test a more direct role Lis1 might play on postmitotic neurons we probed its potential function by conditionally deleting Lis1 in CA1 of the adolescent (P20) hippocampus. Such conditional mutants develop normally during embryonic and early postnatal periods. To generate *Lis1cko* mutants, we crossed *Lis1*^*flox/flox*^ (Hirotsune et al, 1998) mice with the *CamKII-cre* (T29-1) driver line (Tsien et al, 1996; Supporting Information Fig 5). Cre expression in this line is highly selective for the CA1 region of the postnatal hippocampus where recombinase activity is first detected at P19-P20 (Tsien et al, 1996). On a global morphological level we could not detect any cell patterning differences between controls and *Lis1cko* animals in four-week old animals (Supporting Information Fig 4). Golgi-Cox staining of CA1 pyramidal neurons showed that loss of *Lis1* postnatally did not affect dendritic morphology at P28 (Fig 7A and B). However, spine density on secondary and tertiary branches was significantly reduced in *Lis1cko* animals (Fig 7A–C). The Sholl analysis of CA1 pyramidal neurons stained with the Golgi technique confirmed the qualitative impression that there were no significant differences between the two genotypes (Fig 7D–F). To further test the role of *Lis1* in synapse formation and to preclude influences of an earlier developmental history of Lis1 deficiency, we investigated dissociated hippocampal cultures of *Lis1*^*flox/+*^ neurons that were treated with Cre-EGFP lentivirus (Fig 7H and J) or control inactiveCre-EGFP lentivirus (Ho et al, 2006; Fig 7G and I, Supporting Information Fig 5) at DIV1. As in the experiments using *Lis1*^*+/−*^ neurons, we quantified putative synapses by immunostaining for synaptic proteins (PSD95, vGlut1) and GFP to detect infected neurons. Puncta (inserts in Fig 7G–J) where PSD95/vGlut1 immunostaining overlap was obvious were counted as potential synaptic clusters. Once again, we found significant decreases in synaptic cluster numbers at DIV7 and DIV14, whereas by DIV21 synaptic cluster densities appeared to be similar between *Lis1*^*flox/+*^ and *Lis1*^*ko/+*^ neurons (Fig 7K).

### *Lis1cko* mice display deficits in social novelty recognition

Next, we asked whether a Lis1 deficit that begins at P20, is restricted to CA1, and that is associated with loss of hippocampal spines and synaptic cluster formation delay would result in behavioural deficits. We performed the three-chamber social behavioural test and found no differences between adolescent control and *Lis1cko* animals in the sociability aspect where the mice can initiate social contact with a partner/stranger (S1) held in a wire cup or empty (E) wire cup (Fig 7L and M). Both controls and mutants spent more time in the compartment and in close interaction with ‘Stranger 1’ rather than the ‘Empty’ wire cup. Interestingly, *Lis1cko* mutants, unlike controls, showed deficits during social novelty recognition testing in which mice have the opportunity to choose between stranger 1 (S1) and the novel stranger (S2; Fig 7N and O). In contrast to their *Lis1*^*flox/+*^ or *Lis1*^*flox/flox*^ control siblings that preferred the chamber and interaction zone around the novel stranger (S2), *Lis1cko* mice spent similar amounts of time in both compartments and in both interaction zones (Fig 7N and O).

## DISCUSSION

**Figure 8:**
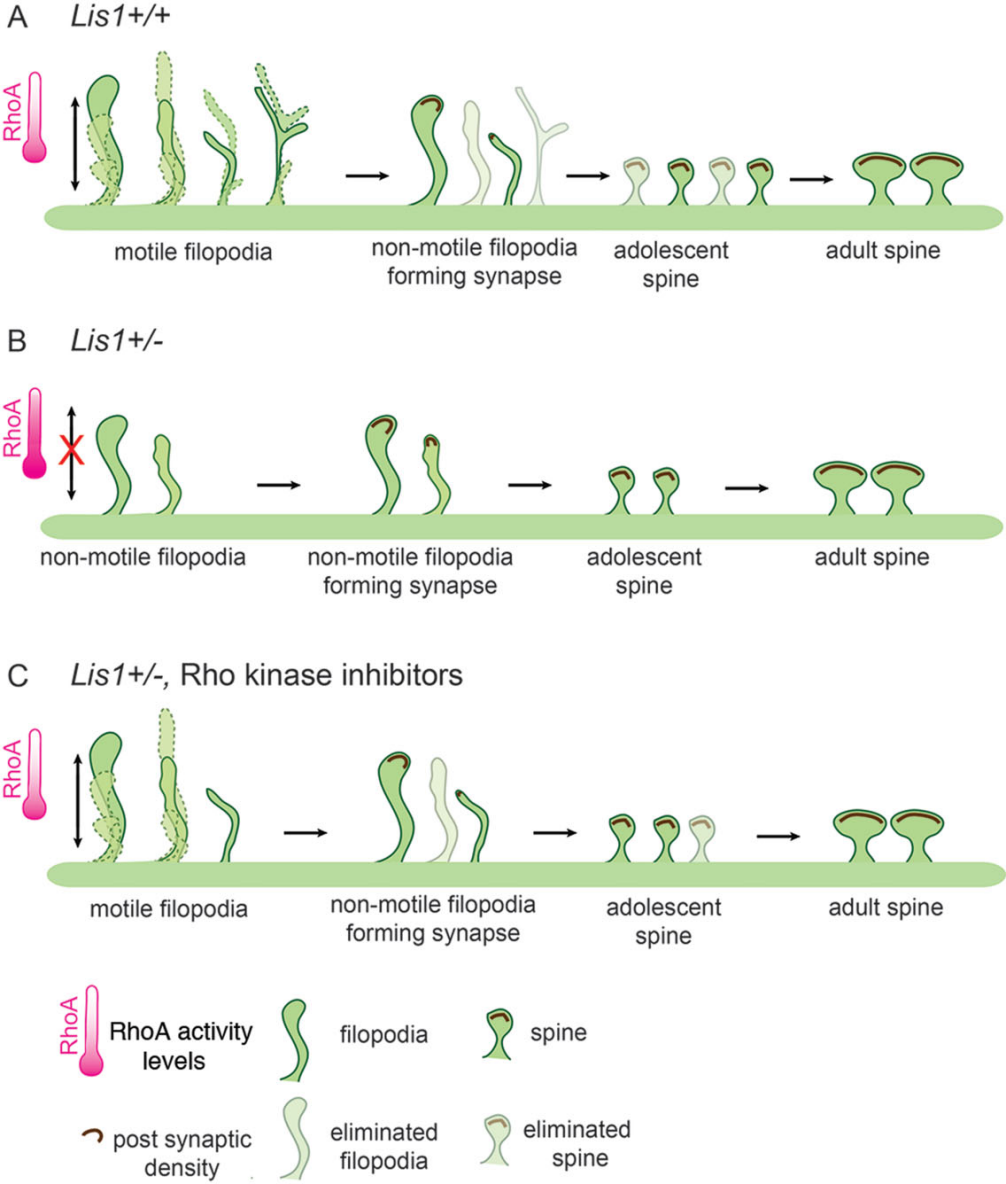
Model: Increased RhoA activity in *Lis1^+/−^* neurons impacts dendritic protrusion motility with consequences for proper circuit formation. A. In *Lis1* wild type neurons, appropriate RhoA levels permit protrusive motility of filopodia, open to making early synaptic connections. Filopodia with weak and non-functional connections get eliminated as part of circuit maturation and refinement. Some of these filopodia with early synaptic connections morph into spines. B. Developing *Lis1*^*+/−*^ neurons exhibit fewer filopodia and elevated GTP-RhoA levels. High RhoA activity impairs filopodial protrusive motility, causing them to be rigid and overly stable, and synaptic connections accumulate more slowly. Overstability prevents elimination and appropriate pruning. Absence of pruning ends in accumulation of dendritic protrusions with synapses, which results in spine densities that are similar between adult mutants and wild types. C. *Lis1*^*+/−*^ neurons treated with Rho kinase inhibitors display restored protrusive motility and length in a subset of filopodia. This allows formation of proper connections and pruning.

This study provides new functional and molecular insight into the postsynaptic role of LIS1 in synaptic plasticity. *In vivo* analysis revealed that both dendritic filopodia and spine dynamics are altered in the adolescent brain of *Lis1*^*+/−*^ mice (Figs 1 and 8). Furthermore, deficits in filopodia length, motility and actin polymerization can be rescued by downregulation of RhoA activity (Figs 2, 3 and 8). We also found a delay in synaptic cluster formation *in vitro*, reduced spine density *in vivo* and deficits in social interactions and social novelty recognition in adolescent *Lis1*^*+/−*^ and *Lis1cko* animals (Figs 4–7). Thus, Lis1 plays a pivotal role in the dynamics of dendritic filopodia and spines during adolescence with an impact on synaptogenesis and, ultimately, behaviour.

Actin dynamics are essential to filopodia and spine plasticity. Approximately 85% of actin is exchanged over a period of minutes in spines (Star et al, 2002). Our data, particularly the results of FRAP experiments (Fig 3) indicate that Lis1 affects dendritic protrusion dynamics through effects on actin polymerization. Previous work demonstrated that a deficiency of proteins that impact the actin cytoskeleton affect the number and activity of synapses, as well as memory and learning (Soderling et al, 2007). Consistent with those studies, we show here for the first time that Lis1 is involved in synapse formation, causing delayed synaptic cluster formation in *Lis1*^*+/−*^ excitatory neurons (Fig 5). Since Lis1 is involved in multiple protein–protein interactions (Kholmanskikh et al, 2006; Vallee & Tsai, 2006), it could modulate a number of signalling events important for synapse formation and maintenance, including influencing other multi-functional scaffold proteins that impact Rho GTPase activities and actin. We also cannot exclude a potential role for dynein on protrusion dynamics, possibly implicated by a recent study of synapse displacement in *Lis1*^*+/−*^ inhibitory neurons (Kawabata et al, 2012). It will be important to characterize signalling pathways in more detail to determine which are the most relevant for Lis1 actions on synaptogenesis and spinogenesis.

Lis1 preferentially affects elimination of filopodia and spines, while their rates of formation are less affected during adolescence in mice (Fig 1). Adolescence is a crucial period, during which adult intellectual abilities, such as multitasking, are formed, and in humans and other mammals is characterized by extensive (∼50%) loss of synapses (Grutzendler et al, 2002; Huttenlocher & Dabholkar, 1997; Markus & Petit, 1987; Rakic et al, 1986; Zuo et al, 2005), achieved by pruning. Deficits in this programmed elimination have been speculated to underlie schizophrenia (Faludi & Mirnics, 2011). However, the molecular mechanisms that govern synaptic maintenance, including proper formation and pruning, remain elusive. Although spine densities of mature CA1 pyramidal neurons in *Lis1*^*+/−*^ adults are similar to those in wildtype animals (Fleck et al, 2000), we found a significantly lower spine density in adolescent mutant animals, together with a reduced rate of spine turnover. Interestingly, 24 h after systemic injection of ROCK inhibitors into *Lis1*^*+/−*^ adolescent animals neurite spine density was restored to control levels, further supporting a role of RhoA hyperactivity in this deficit. Therefore, *Lis1* haploinsufficient neurons are impaired in the pruning of dendritic spines, so that they continue to steadily accumulate spines throughout adolescence, and by adulthood finally reach the same density as wild type neurons (Fig 8). Failure to prune protrusions in *Lis1* deficiency could overstabilize ‘adolescent’ spines, producing defective synapses and an adult neural network with reduced capacity for synaptic plasticity. Furthermore when *Lis1* was conditionally deleted from CA1 pyramidal neurons at P20, analyses at P28 showed significant decreases in spine densities on secondary and tertiary dendritic branches of *Lis1cko* mice compared to controls. This *CamKII:Lis1cko* model allows one to isolate the post-mitotic, post-migrational role of Lis1 in neurons because gene deletion does not occur until P20, after neuronal position and dendrite arbours are established. Moreover, this conditional knockout results in a complete loss of *Lis1* in adolescent hippocampus in contrast to *Lis1*^*+/−*^. These experiments support the hypothesis that impaired development of neural networks may underlie the deficits in social behaviours in adolescent *Lis1*^*+/−*^ and *Lis1cko* animals demonstrated here, and later in life in learning and memory (Paylor et al, 1999). Indeed, while *Lis1*^*+/−*^ animals exhibited deficits in both paradigms of the three-chamber social behaviour test, *Lis1cko* mice had a distinct defect in memory retrieval as evidenced by deficits in social novelty recognition. Behavioural testing results of the *Lis1cko* are fitting as hippocampus is responsible for learning and memory and the structure has been implicated in patients with ASD (DeLong, 1992).

Neurodevelopmental disorders such as ASD, schizophrenia, and epilepsy have been associated with disturbances in local neural networks. Furthermore, the majority of the recently identified ASD candidate genes encode proteins found in excitatory synapses (Toro et al, 2010), suggesting that these disorders may arise from synaptic dysfunction. Mutations of glutamatergic postsynaptic proteins *ProSap2/Shank3* and *ProSap1/Shank2* in mice lead to distinct disturbances in excitatory synapses and profound defects in social interaction behaviour (Bozdagi et al, 2010; Peca et al, 2011; Schmeisser et al, 2012). The modelling in mouse of human neurodevelopmental disorders is crucial for investigation of disease mechanisms at molecular and cellular levels, and for trials of potential pharmacological treatments. The distinctive phenotype affecting social interactions without repetitive behavioural deficit make *Lis1*^*+/−*^ mice an attractive model for developing therapeutic treatments targeted to social aspects of behavioural disorders, especially those intersecting with Lis1, as recently implicated in ASD (Neale et al, 2012).

In conclusion, we demonstrated that *Lis1* plays a crucial role in cortical circuit assembly by regulating filopodia and spine turnover to establish correct connections and promote synaptic plasticity during adolescence in mice. These dynamics could easily be overlooked in mutant mouse models that are typically assessed only by dendritic spine density in adults. Furthermore, we showed that while Lis1 plays a major role throughout development it has a separate potent and crucial role in adolescent neurons. Our results provide a novel path through LIS1 for investigation of the connectivity and plasticity of post-migrational neurons. They also suggest that LIS1 could make an important therapeutic target for intervention in developmental neurobehavioural disorders like ASDs and schizophrenia, and provides a manipulable model for one of the cardinal features of these disorders, namely impaired socialization.

## MATERIALS AND METHODS

### Mice

Animals were housed at a constant temperature of 23°C with a 12 h light/dark cycle, with food and water *ad libitum*. All protocols involving mice were reviewed and approved by the Institutional Animal Care and Use Committee. The following mouse lines were used and genotyped as described previously: *Lis1*^*flox*^ (Hirotsune et al, 1998), *Lis1*^*+/−*^ (Hirotsune et al, 1998) and *CamKII-Cre* (Tsien et al, 1996). The day of birth was designated as postnatal day 0 (P0). We considered P20–P30 as adolescent and P60 as adult animals.

#### Histology and immunohistochemistry

Animals received an intraperitoneal injection of Ketamine/Xylazine and when fully anaesthetized were transcardially perfused with 20–30 ml of PBS followed by 60 ml of 4% paraformaldehyde. Dissected brains were postfixed for 2–4 h, cryoprotected in 30% sucrose, and stored at 4°C until sectioning on a cryostate at 40 µm and serial sections were collected into 96-well plates containing 0.05% sodium azide, stored at 4°C. Immunohistochemistry performed on free-floating sections under standard staining procedures used the following primary antibodies: mouse anti-NeuN (Millipore) and rabbit anti-Lis1 (Abcam). Species-specific, biotinylated secondary antibodies (Vector) were used at 1:400 dilution followed by incubation in ABC (avidin–biotin comples) (Vector). Visualization was achieved using diaminobenzidine (Vector) as developing agent.

#### Immunohistochemical image acquisition

All images were taken using an upright compound microscope (Nikon, Japan), acquired with a digital camera and Spot Insight Mosaic 3.2 (Diagnostic Instruments, USA) and processed using Adobe Photoshop. Images were not modified in any way, except for adjustments of brightness and contrast. All quantitative data were compiled in MS Excel and significance was evaluated by the Student's *t*-test.

### *In vivo* transcranial two-photon imaging and analysis

A Bio-Rad Radiance 2000 two-photon microscope was used for imaging dendritic protrusions in the mouse barrel cortex. Images were taken with a 60X objective and 1.5X zoom. The degree of spine formation and elimination was obtained from longitudinal studies by imaging the mouse cortex through a thinned-skull window. *Thy1-YFP*;*Lis1*^*+/+*^ and *Thy1-YFP*;*Lis1*^*+/−*^ mice age P21, P23, P30 and P44 were imaged. Specifically, the ‘10 min’ interval of filopodia dynamics represented scanned images at P21, taken every 10 min over a 1 h period (Supporting Information Fig 1A). The 1 h ‘P21’ interval (Fig. 1C) mice were imaged so that scan/view 1 was taken at 0 h, followed by scan/view 2 1 h later. The 48 h interval (‘P21–P23’; Fig. 1D) quantified filopodia dynamics first in scan/view 1 taken at P21 compared with scan/view 2 at P23. Regarding spine dynamics, 48 h ‘P21–P23’ (Fig. 1E) compared imaging of spines first performed at P21 (scan/view 1) followed by scanning of the same region at P23 (scan/view 2). Finally, 14 days ‘P30–P44’ (Fig. 1F) spine imaging was first performed at P30 (scan/view 1) followed by the second scan of the exactly same region 14 days later at P44 (scan/view 2). Technical procedures are detailed elsewhere (Yang et al, 2010). ImageJ software was used to analyse image stacks. Data analysis was performed as previously described (Yang et al, 2009). Briefly, the same dendritic segments were identified from three-dimensional stacks taken from different time points with high image quality. The total numbers of filopodia or spines (*n*) were pooled from dendritic segments of different animals. Spines or filopodia were considered the same between views if their positions remained the same distance from relative adjacent landmarks. Spines were considered different if they were more than 0.7 µm away from their expected positions based on the first view. The term ‘turnover’ of filopodia and spines is used to describe how often a filopodium or spine is present and then lost during the time of imaging. It represents the sum of the percentage of all filopodia that are formed and eliminated during imaging period (Trachtenberg et al, 2002). It is calculated as (*N*_gained_ + *N*_lost_)/(2 × *N*_total_). Conversely, ‘stability’ is a term used to describe stable or constant presence of a protrusion (spine or filopodium) during the time of imaging. Spines or filopodia were identified as stable if they were present in scan/view 1 and scan/view 2, eliminated if present in scan/view 1 but not in scan/view 2 and formed if present in scan/view 2 but not in scan/view 1.

### Definition of protrusions

Filopodia are long and thin protrusions, whereas spines are shorter with clear enlargements termed the ‘head’ of spines, or smaller stubby looking protrusions. Filopodia were identified as long, thin structures (generally larger than twice the average spine length, with a ratio of head diameter to neck diameter <1.2:1 and ratio of length to neck diameter >3:1). The remaining protrusions were classified as spines. No subtypes of spines were separated.

### Cell culture

P0–P1 hippocampal neurons from *Lis1*^*+/+*^ or *Lis1*^*+/−*^ mice were cultured using established procedures (Kholmanskikh et al, 2006). Neurons were imaged in recording media (L15 medium, 10% foetal bovine serum, 0.5% glucose). Cultures were incubated for 4 h in the presence of Rho-kinase inhibitor 10 µM Y27632 (Enzo LifeSciences) or with the drug vehicle and then fixed to examine filopodia length rescue. For filopodia motility dissociated hippocampal neurons were incubated in the presence of 10 µM HA1079 and 10 µM Y27632 (Enzo LifeSciences) for 1 h prior to imaging.

### Spinning-disc confocal imaging

Images were captured using an inverted 200M Zeiss microscope connected to a laser confocal scanning head (UltraView, Perkin-Elmer). Objective and stage are encased in an incubation chamber (Zeiss), where +37°C temperature is maintained. Three-dimensional images were reconstructed using MetaMorph software (Universal Imaging).

### Quantification of filopodia dynamics *in vitro*

Images were captured on 200M Zeiss spinning-disc confocal microscope with 63X objective at intervals of 2 min over a 20 min period for a total of 11 images. We analysed 10–15 protrusions per neuron within 50 µm from the cell body. The regions were randomly chosen as long as we could visualize the cell body and follow the dendrite. Images were analysed for changes in tip position and protrusion length. The protrusion tip in each frame was marked and then each image of the recording was overlaid to denote protrusion motility. Motility values estimated movement magnitude calculated as the accumulated length change divided by the duration of observation (*M* = ∑[*x*_*n*_ − *x*_*i*_]/20; where *x*_*n*_ is the length for protrusion *x* in frame *n*, and *x*_*i*_ is the length for the same protrusion in the first frame; Brocco et al, 2010; Tashiro & Yuste, 2004). The LMI specifically measures the extension–retraction motility (Brocco et al, 2010; Tashiro & Yuste, 2004). For calculating the LMI, the smallest length was subtracted from the maximum length and divided by the average length of the protrusion. LMI values for control neurons were normalized to 1.

### FRAP

Dissociated hippocampal neurons from P0–P1 pups were cultured on poly-D-lysine and laminin coated glass bottom dishes (MatTek). Neurons were infected with AAV vector containing GFP-Actin monomer 4 h post plating. AAV vectors were constructed as described (Morgenstern et al, 2011). Briefly, virus stocks were prepared by packaging the vector plasmids into AAV serotype 2 particles with a helper-free plasmid transfection system. The vectors were purified with heparin affinity chromatography and dialyzed against PBS. AAV titers were determined by qPCR. Neurons were used for FRAP experiments at DIV12-14. Both filopodia and spines were used for quantification, however the majority of protrusions were spines due to the stage of the hippocampal culture. The culture medium was exchanged with prewarmed recording media containing: L15 media, 10% foetal bovine serum, 0.5% glucose. Temperature was kept at 37°C using a heated chamber. For rescue experiments dissociated hippocampal neurons were incubated in the presence of 10 µM HA1079 and 10 µM Y27632 (Enzo LifeSciences) for 1 h prior to imaging. All images were randomly chosen and captured using an inverted Olympus IX-70 microscope using a 100X oil objective. Photobleaching was achieved by 488 nm laser transmission output set at 50%. A series of images was captured before and immediately after photobleaching. Three images were captured before bleaching and their average fluorescence was normalized to 1. Subsequent images were obtained at 0.5 s intervals over a period of 180 s. ImageJ software (NIH) was used for aligning the images and measuring fluorescence intensity of the region of interest within time-lapse videos. For each movie, the mean intensity of an untransfected area was measured as background and was subtracted from the original intensity. Bleached areas used for measurements were outlined to contain only the protrusion. For each genotype three separate dissociated cultures were set up. The values from different protrusions were averaged and the mean values were charted in a scatter plot.

### Immunocytochemistry

Neurons were fixed for 10 min in 4% paraformaldehyde in PBS at 4°C. After blocking in PBS containing 0.3% Tween/10% normal donkey serum for 1 h at room temperature, cells were triply stained with primary antibodies for 2 h at room temperature and incubation with secondary antibodies for 1 h in the dark. Primary antibodies used were anti guinea-pig vGlut1 (Milipore), anti-mouse PSD-95 (Abcam), anti-rabbit MAP2 (Sigma), anti-rabbit TuJ1 (Covance), anti-rat GFP (Nacalai Tesque). Secondary antibodies used were AlexaFluor488 goat-anti guinea pig, AlexaFluor488 goat-anti rat, AlexaFluor568 donkey-anti mouse, AlexaFluor647 donkey-anti rabbit, AlexaFluor568 donkey-anti rabbit (Molecular Probes). For quantification of synaptic clusters, a stack of optical sections (Z step, 0.5 µm, on average 5 steps/region) was acquired using a 63X objective on 200M Zeiss spinning-disc confocal microscope. The optical sections were processed for maximum projection and analysed. Only puncta where there was a clear overlap between vGlut1 and PSD-95 were considered potential synaptic clusters. Quantifications were performed using ImageJ.

### Golgi staining

Brains from 3-week old (P21) *Lis1*^*+/+*^, *Lis1*^*+/−*^ littermates and 4-week old (P28) *Lis1*^*flox/+*^, *Lis1cko* mice were processed in parallel (*n* = 3) and sections were stained using a modified Golgi-Cox impregnation of neurons following the manufacturer's protocol (FD NeuroTechnologies). For spine density quantification, the number of spines along equivalent lengths of dendritic segments proximal to the cell body was counted on pyramidal neurons from the CA1 region of hippocampi of *Lis1*^*+/+*^, *Lis1*^*+/−*^, *Lis1*^*flox/+*^ and *Lis1cko* mice. Images were captured with a 100X objective on a 200M Zeiss spinning-disc confocal microscope with brightfield settings. Postnatal day 20 *Lis1*^*+/+*^ and *Lis1*^*+/−*^ mice were injected intraperitoneally with either Y-27632 (10 mg/kg, Tocris Cookson LTD, UK) reconstituted in 0.9% normal saline or saline alone. Twenty-four hours after Y-27632 or control treatment mice were processed for Golgi staining (P21). The drug treatment followed a previously published protocol (Jeon et al, 2012). In the treated animals, we found that of all quantified protrusions (∼90%) appeared to be spines.

**The paper explained**

**PROBLEM**

In humans, a 50% loss of LIS1 causes lissencephaly as a result of aberrant neuronal migration and organization. Interestingly, LIS1 is present in post-migrational neurons and their synapses. Furthermore, LIS1 has been associated with the interaction networks of high risk Autism Spectrum Disorder genes. However, the role of LIS1 remains poorly understood in the aetiology of this and other disorders associated with altered synapse formation and pruning.

**RESULTS**

Using *in vivo* and *in vitro* imaging of neuronal processes in a mouse model lacking one *Lis1* gene copy we demonstrate a prominent Lis1 role in the dynamic behaviour of protrusions important for synapse formation and maturation. Reduced protrusion dynamics are associated with delayed synapse formation in *Lis1*^*+/−*^ neurons. Finally, *Lis1*^*+/−*^ and *Lis1cko* mutant mice showed deficits in social interactions, establishing a link between *Lis1* gene function and autistic-like behaviours in mice.

**IMPACT**

The role of Lis1 in the regulation of proper formation and elimination of connections between neurons is an important new avenue for investigation of mechanisms contributing to neurobehavioural disorders in which altered socialization is a prominent component. Because Lis1 action is exquisitely gene-dosage dependent, it is an attractive target for development of pharmacological therapies.

### Sholl analysis

Neurons selected for analysis were imaged using a 40X objective on a Zeiss microscope and used for the Sholl analysis (Sholl, 1953) of dendritic complexity. The neurons were traced using Neurolucida software (MicroBrightField), and the Sholl analysis used Neuroexplorer software (MicroBrightField) to calculate the cumulative umber of dendritic intersections at 10 µm interval distance points starting from the cell body. The analysis of the number of dendrite intersections was performed separately for apical and basal dendrites.

### Lentivirus production

Lentiviral vectors used were pFUGW EGFP-NLS-CRE driven by a ubiquitin promoter and pFUGW EGFP-NLS (cre mutant) control (Ho et al, 2006). Lentivirus production was performed as described previously (Marongiu et al, 2009; Tiscornia et al, 2006). *Lis1*^*flox/+*^ dissociated hippocampal cultures were infected with lentiviruses at DIV1. Analysis was performed at DIV7, DIV14 and DIV21.

### Western blot

Cell lysates were prepared from P0–P1 dissociated hippocampal neurons, cultured for 1 day before being exposed to Cre or ΔCre lentivirus for an additional 13 days and collected at DIV14. Protein lysates for the CA1 region were prepared by placing P28 *Lis1*^*flox/+*^ and *Lis1cko* whole hippocampi into cold PBS, then dissecting the approximate CA1 region for lysis of tissue. Protein levels were analysed on Western blots using rabbit anti-IQGAP1 (Santa Cruz) as a loading control and mouse anti-Lis1 (Sigma–Aldrich). The protocol used was described previously (Kholmanskikh et al, 2003, 2006).

### Social interaction paradigm

Three-chamber social test: sociability and response to social novelty was tested as described previously (Moy et al, 2004; Silverman et al, 2010) with minor modifications. Briefly, 4 week old male animals were used across all tests for *Lis1*^*+/+*^ and *Lis1*^*+/−*^ and both genders were used for *CamKII:Lis1cko* tests. Test mice were habituated to the testing room for at least 1 h before the start of behavioural tasks. The social test apparatus consisted of a white acrylic box with removable transparent partitions dividing the box into three chambers. The dimension of each chamber was 22 cm × 43 cm, whereas the wire cups used to contain the stranger mouse were cylindrical, 9 cm in height with a bottom diameter of 8 cm with the mesh spaced at 0.5 cm apart. An inverted transparent cup was placed on the top of the wire cup to prevent the test mice from climbing on the top of the wire cup. For the sociability test, the test animal was introduced to all three chambers for 5 min. After the test mouse was secluded in the middle chamber, Stranger 1 was placed in either the left or right chamber and dividers were then raised, allowing the test animal to freely explore all three chambers over a 5 min session. Following this, the test animal was secluded in the middle chamber, Stranger 2 was placed in a previously empty cup, dividers were raised and the test animal was allowed to freely explore all three chambers for a 5 min session. Time spent in each chamber and time spent in close proximity (termed as time in close interaction) to the wire cup were calculated using automated Anymaze software (Stoelting Anymaze). The close interaction with the cup or stranger in the cup was determined if the subject mouse was facing the cup and its nose was physically touching the cup (presumably sniffing).

### Marble burying

Young adult animals (P32–P35), including males and females, were used for this test. Clean cages with 4 cm of bedding were prepared with 12 evenly spaced blue glass marbles. Testing consisted of a 30 min exploration period. The number of marbles buried (>50% marble covered by bedding material) was recorded.

### Olfactory habituation/dishabituation

Testing was conducted on male and female *Lis1* wild-type and heterozygous mice aged 2–4 months. Non-social and social odours were presented on a series of cotton swabs inserted into the home cage sequentially, each for 2 min, in the following order: water, water, water (distilled); almond, almond, almond (1:100 almond extract); banana, banana, banana (1:100 banana extract); social 1, social 1, social 1 (male urine); social 2, social 2, social 2 (female urine).

### Statistical analyses

All image data were analysed with the operator blinded to genotype. At least three independent trials were performed for each video microscopy experiment and the numbers of observations are given throughout the text and figure legends. Statistical significance of the difference between mean values was determined using a two-tailed Student *t*-test. For all data error bars indicate s.d. The standard Student's *t*-test was used to determine the statistical significance of results for FRAP experiment. The Sholl analysis data were tested by applying a two-tailed Student *t-*test point by point.

## Author contributions

AS and MER designed all experiments; FG and WBG designed two photon experiments. AS and FG performed two photon experiments; AS and DT performed behavioural experiments; AS and MER analysed data and wrote the manuscript; All of the authors contributed to the final version of the manuscript.

## Notes

As a service to our authors and readers, this journal provides supporting information supplied by the authors. Such materials are peer reviewed and may be re-organized for online delivery, but are not copy-edited or typeset. Technical support issues arising from supporting information (other than missing files) should be addressed to the authors.
