# Supplementary material for: Lis1 controls dynamics of neuronal filopodia and spines to impact synaptogenesis and social behaviour
Source: EMBO Mol Med. 2013 Mar 11;5(4):591–607. doi: 10.1002/emmm.201202106 (PMC3628102; doi:10.1002/emmm.201202106)
Supplement: Supplementary file 1 [file emmm0005-0591-sd1.pdf]

EMBO Molecular Medicine

Lis1 controls dynamics of neuronal filopodia and spines to impact synaptogenesis and social behaviour

Anamaria Sudarov, Frank Gooden, Debbie Tseng, Wen-Biao Gan, Margaret Elizabeth Ross

Corresponding author: Margaret Ross, Weill Cornell Medical College

Review timeline:

Submission date:	08 August 2012
Editorial Decision:	13 August 2012
Additional Author Correspondence:	14 August 2013
Additional Author Correspondence :	14 August 2012
Resubmission:	01 October 2012
Editorial Decision:	06 November 2012
Revision received:	20 December 2012
Editorial Decision:	20 January 2013
Revision received:	29 January 2013
Accepted:	31 January 2013

Transaction Report:

(Note: With the exception of the correction of typographical or spelling errors that could be a source of ambiguity, letters and reports are not edited. The original formatting of letters and referee reports may not be reflected in this compilation.)

Editor: Anneke Funk / Roberto Buccione

1st Editorial Decision 13 August 2012

Thank you for the submission of your manuscript "Lis1 controls dynamics of neuronal filopodia and spines to impact synaptogenesis and social behavior". I have now had the opportunity to carefully read your paper and the related literature and I have also discussed it with my colleagues. I am afraid that we concluded that the manuscript is not well suited for publication in EMBO Molecular Medicine and have therefore decided not to proceed with peer review.

The manuscript reports that Lis1 is important for filopodia and spine turnover. Using adolescent heterozygous Lis1 KO mice, you show deficits in filopodia dynamics as well as increased stability and decreased elimination rates of dendritic spines in addition to reduced spine density in CA1 pyramidal neurons. In cultured hippocampal neurons from these mice, filopodia density and motility are reduced, which can partially be rescued by RhoA inhibition. In addition, you show that the Lis1-deficient neurons display actin turnover defects in dendritic protrusions. In vitro, you show that synaptic cluster formation is delayed in Lis1 +/- hippocampal neurons. In addition, you show that the Lis1-deficient mice display reduced social interaction and abnormal social novelty recognition.

We appreciate that you suggest that Lis1 plays a postsynaptic role in synaptic plasticity by regulating filopodia and spine turnover, which affect social behaviour. As such, we do recognize the potential interest of the findings. However, at this stage, we feel that the study does not provide the sort of mechanistic insight we would expect in an EMBO Molecular Medicine article. Specifically, it remains unclear whether the observed detrimental effects on filopodia and spine turnover indeed are causally linked to the described behavioural phenotype of the Lis1 +/- mice.

© EMBO

Given the potential interest of the findings, we would, however, have no objection to consider a new manuscript on the same topic if at some time in the near future you obtained data that would considerably strengthen the study by providing evidence for a causal role of Lis1-regulated filopodia and spine turnover or synaptogenesis in the behaviour of the studied mice.

I am sorry that I could not bring better news this time but hope that this negative decision does not prevent you from considering our journal for the publication of your future studies.

Additional Author Correspondence 14 August 2013

Thank you for your most thoughtful communication. My colleagues and I are encouraged that the Journal appreciates the potential interest of this work. We would be most interested in attempting to provide additional evidence for a causal role of Lis1-regulated filopodia and spine turnover or synaptogenesis in mice deficient in Lis1.

An experiment that we are prepared to undertake is to generate conditional Lis1+/- mice that would inactivate Lis1 only in hippocampus starting at around postnatal day 20 and test these animals in this socialization paradigm. This would have the advantage that Lis1 would be inactivated after both embryonic neurogenesis and developmental neuronal migration are complete. Behavioral testing would begin within 2 weeks after the onset of Cre expression. Although it is not possible to limit Lis1 loss to the synapse in the intact system, this conditional knockout would be the closest approximation we can think of in a behavioral paradigm to allow examination of Lis1 effects in established neurons.

Should we be successful in this experimental approach, would such data potentially address the journal's concerns?

Additional Author Correspondence 14 August 2012

Thank you for your message and your continued interest.

I considered your experimental plan and agree that it would significantly strengthen the study. Thus, I would be glad to receive an extended manuscript containing the mentioned experiments in due time.

Resubmission 01 October 2012

Enclosed, please find an extended manuscript entitled “Lis1 controls dynamics of neuronal filopodia and spines to impact synaptogenesis and social behavior,” by Sudarov et al., which is being submitted in consideration for publication in EMBO Molecular Medicine.

The manuscript uncovers a novel role for Lis1 in synaptogenesis and neurodevelopmental behavior. This is particularly important as recent reports, including Neale et al. (Nature 2012, 485:242), have implicated LIS1 protein as a hub in the networks of high-risk genes associated with autism spectrum disorder. However, there is little information regarding how LIS1 might participate in such neuro-behavioral disorders, especially in the absence of cortical malformation demonstrable on MR imaging. Leading hypotheses regarding autistic behaviors include not only formation of faulty synaptic contacts but also failure to successfully prune synapses once formed or make new connections during childhood development. In this report, transcranial two-photon live imaging shows that Lis1+/- mouse brains in vivo and their dissociated neurons in vitro display just such defects. Our results link defects in the dynamics of filopodia and spines to synapse formation, plasticity and contribution to autistic-like behavior in mice.

An earlier version of the manuscript was submitted to your journal in mid August, 2012. At that time, the Editors' main concern was that there should be evidence provided that Lis1 deficiency has a primary role in synaptogenesis and plasticity, rather than a secondary impact due to earlier developmental events. We now have such evidence reported in 3 new experiments (new Figure 7) that take advantage of the mouse bearing a Lis1-floxed allele that allows for conditional inactivation of the gene. First, dissociated Lis1flox/+ hippocampal neurons infected with a lentivirus expressing GFP-Cre recombinase to acutely inactivate Lis1 exhibited a delay in synaptic cluster formation at DIV7 that caught up by DIV21. Second, Lis1flox/+ animals were bred with the CamKIICre line to inactivate Lis1 only in the CA1 region of the hippocampus (Lis1cko), starting at postnatal day 20 (P20). While gross hippocampal morphology in P28 Lis1cko animals was normal, Golgi silver impregnation revealed reduced spine densities on CA1 neurites, just as observed in the Lis1+/– neurons. Third, social interaction behavior was tested in the CamKIICre:Lis1flox/flox conditional knockout animals at P28, using the same 3-chamber paradigm employed in studies of the Lis1+/– model. Interestingly, while the first part of the test was no different between mutants and controls, the Lis1cko animals displayed significant deficits in social novelty recognition—the more hippocampus dependent arm of the test.

This manuscript provides new insight into the connection between filopodia and dendritic spine dynamics and synaptic pruning, which could easily be overlooked in mutant mouse models that are typically assessed only by dendritic spine density in adults. It also suggests a potentially important therapeutic target for intervention in developmental neurobehavioral disorders, and provides a manipulable model for one of the cardinal features of these disorders, namely impaired socialization. My colleagues and I are very excited with these additional results and believe that they greatly strengthen the manuscript. We hope that these added data will increase your interest in the report and that you will send it out for expert review.

2nd Editorial Decision 06 November 2012

Thank you for the submission of your manuscript to EMBO Molecular Medicine. We have now heard back from the three Reviewers whom we asked to evaluate your manuscript.

Although the Reviewers find the topic of the study potentially interesting and important, they also raise a number of substantial concerns about the interpretation and conclusiveness of the data and technical execution of the experiments. As you will see from the enclosed reports, a number of critical experiments are required to fully support your conclusions.

Reviewer 2 recognises the technical difficulty of many experiments, but is especially concerned with the fact that it is unclear how certain experiments were exactly performed. S/he suggests specific experimental approaches and improvements to increase clarity and confidence in the data.

Reviewer 3 shares similar concerns and emphasizes that some important claims are not fully supported by definitive data. For instance, the statement that conditional Lis1 knockout in adulthood does not affect dendritic structure is not supported by data. Finally, Reviewer 3 also feels that your work does not provide sufficient mechanistic insight into how Lis1 affects cell morphogenesis. I do appreciate, however, that the main emphasis of your work is on linking Lis1 function and psychiatric disorders. Thus, I would be satisfied if all other concerns are carefully dealt with.

On the more clinical side, Reviewer 1 raises an issue with the correct interpretation of the pathophysiological implications of your study, which also needs to be addressed.

While it is clear that publication of the paper cannot be considered at this stage, I am open to the submission of a revised manuscript, provided however, that the Reviewers' concerns are fully addressed.

I should remind you that it is EMBO Molecular Medicine policy to allow a single round of revision only and that, therefore, acceptance or rejection of the manuscript will depend on the completeness of your responses included in the next, final version of the manuscript.

As you know, EMBO Molecular Medicine has a "scooping protection" policy, whereby similar findings that are published by others during review or revision are not a criterion for rejection. However, I do ask you to get in touch with us after three months if you have not completed your revision, to update us on the status. Please also contact us as soon as possible if similar work is published elsewhere.

I look forward to seeing a revised form of your manuscript.

***** Reviewer's comments *****

Referee #1 (General Remarks):

This is a well written paper and experimentally well-planned set of studies detailing a connection between Lis1 and regulation of spines in synaptogenesis and behavioral control in mice.The following are my comments on this paper:1-There are different descritions of adolescent age in rodents which has implications on correct analysis of the data and its possible association with autism.Most rodent models of autism use P7-P14 as important times for appearance of autistic-like behavior which correspond to ages 1-3 years in human brain development.Results presented here are reflective of adolescence(P20-P30) when most symptoms of schizophrenia occur i.e., 14-16 in male patients.Thus while the results obtained in these studies are novel they have more relevance to schizophrenia than autism ;2-are the dendritic spines described in page 5 similar to long immature dendrites observed in brains of subjects with fragile X syndrome ?

Referee #2 (Comments on Novelty/Model System):

The reason I gave the technical quality a medium rating is that it was difficult to assess the potential for error in many of the experiments. Some controls were not clearly described, and the details of many of the experimental procedures were fairly vague or scattered throughout the manuscript and difficult to follow. The experiments described are difficult and require substantial skill and an strong sense of the possible artifacts that might arise.

Referee #2 (General Remarks):

The manuscript by Sudarov et al proposes that reduced Lis1 expression impacts dendritic protrusions (filopodia and spines). They make the argument that Lis1 loss results in less dynamic protrusions, and that this is linked to elevated RhoA activity.

The data in this paper represents a substantial effort, and these are complex and difficult experiments. The study presents potentially very important findings elucidating a role for Lis1 in the adolescent and adult brain (beyond its clear role in embryonic brain). In particular the behavioral defects are notable and interesting, and have implications for disorders like autism and schizophrenia.

The main concern with this manuscript is that it is sometimes difficult to determine exactly how experiments were done, and hard to gauge the potential for error in the analyses. Most of the confusion can be eliminated with better clarification/organization. I have indicated some ways that would make for an easier read and increase confidence in the findings.

Figure 1:
a) Readers are directed to an article that is not easily accessible for methods of the in situ imaging (Yang et al., 2010). More details should be provided in the methods section. Also, the methods for calculating filopodia motility were not easy to follow, and it was not clear if the same procedures were used in the brain and in cultures.

b) In the introduction the authors indicate that while much is known about regulation of dendritic spine dynamics, less is known about regulation of dendritic filopodia. How are spines differentiated from filopodia in the experiments described here? Are all highly dynamic protrusions considered

filopodia regardless of the age of the animals or cultures? What is the distinction between turnover (1C) and stability (1D-F)?

c) The time lapse intervals, duration of imaging, and time between imaging sessions for the four graphs in this figure need to be more clearly defined, either in the text, the legends, or the methods. It takes a lot of effort on the part of the reader to try to elucidate the relevant specifics for each set of data.

d) Were there fewer filopodia/protrusions in general, as was observed in hippocampal cultures (Fig 2)?

Figure 2:
a) The cells in A and B look like different types of cells. B looks more like an immature neuron, a glial cell, or some other type of neuron rather than a hippocampal pyramidal neuron. (see Benson et al, J. Neurocytology, 1994 May;23(5):279-95).

b) Where along the dendritic shaft were time-lapse sequences obtained? Were the regions randomly selected? What length of dendritic shaft was used for these studies? Are there differences in the density of protrusions/length? If dendrites are longer, there could be equal numbers of protrusions overall, but more sparsely positioned. This would still be interesting but may reflect an alteration in dendritic growth rather than spine dynamics.

c) The legend for D indicates the data are from both 2DIV and 10DIV, but the graph only shows two bars (Lis1+/+ and Lis1+/-).

d) There is confusing reasoning in the text describing the RhoA experiments: "Because Lis1 haploinsufficiency leads to elevated RhoA activity, and constitutive activation of RhoA negatively regulates spine formation and stability, we asked whether upregulated RhoA is responsible for diminished filopodial motility in Lis1+/- neurons". If overactive RhoA prevents spine formation and destabilizes spines, why would the same event reduce motility? This needs to be clarified, or the relationship between motility and stability needs to be clarified.

e) It seems odd that ROCK inhibitors would have no impact on filopodia in control neurons. Is RhoA not involved in preventing filopodial motility under normal circumstances?

Figure 3:
a) Are there error bars for the data in 3D?
b) Is the data statistically significant?
c) How does actin polymerization rate contribute to actin turnover?
d) Was turnover measured in spines or filopodia?

Figure 4:
E and J should be labeled CA1 and barrel cortex to avoid confusion.

Figure 5:
a) Are the images confocal images? If not which of the Z-steps was used for analysis of overlap?
b) It would possibly be more informative if the numbers of vGlut1+/PSD95- and vGlut1-/PSD95+ puncta were included in the analysis.
c) Defects in localization of PSD95 or vGlut1 rather than altered spine behavior could explain this result.

Figure 6:
a) How do the mice spend time "in close interaction" with an empty cup? This is probably a simple thing, but could use more explanation.
b) If possible, it would be interesting to inhibit RhoA to determine if the behaviors are rescued.

Figure 7:
a) There should be some way of determining whether Lis1 mRNA or protein levels were in fact reduced in the intact hippocampus and in the cultured neurons. This might require is situ

hybridization or immunostaining. Western or northern blots might suffice for cultures.

Discussion:

Confusing concept again:

"...we found a significant decrease in spine density in adolescent mutant animals, together with a reduced rate of turnover. Therefor, Lis1 haploinsufficient neurons fail to undergo adolescent pruning of dendritic spines and continue to steadily accumulate spines throughout adolescence, finally by adulthood reaching the same density as wild type neurons."

If there is no increase in the formation of spines, but there is reduced elimination as suggested by Figure 1, why would spine density be lower in mutant adolescent animals? Wouldn't the density increase, because spines are not being eliminated as efficiently? The fact that there is a decreased spine density suggests that there is a defect in spine formation or maintenance.

Referee #3 (Comments on Novelty/Model System):

The work is very descriptive and does not provide important new mechanistic insights into how reduced function of Lis1 controls dendritic spine and dendrite formation.

My recommedation below is really at the bottom end of the enthusiasm range. It would need very significant additional work to reach the profile one would expect for EMBO Mol Med.

Referee #3 (General Remarks):

In this manuscript, Sudarov et al. characterize the cellular and anatomical defects that result from loss or reduced function of the Lis1 protein. Transcranial imaging is used to shown that dendritic filopodia in apical dendrites of layer V barrel cortex neurons are much less dynamic in formation and turnover in Lis1+/- animals during both adolescence and in early adulthood. Moreover, the extension and retraction of individual filopodia is greatly reduced in Lis1+/- neurons relative to wt controls. The author demonstrate that inhibition of ROCK (and not RhoA as stated in the section title) increases filopodial motility. ROCK inhibitors also appears to modestly resuce defects in actin dynamics, although it is hard to gauge the magnitude of this effect based on the spare experimental details given. Lis1+/- mice also have reduced dendritic spine density. The claim is made that hippocampal dendrite arbors are altered in the Lis1+/- mice, but the descriptions are not backed up with rigorous measurements. Similarly, the claim is made that conditional knockout of Lis1 in adulthood does not affect dendrite structure, but this is not backed up with data. The authors go on to show that the Lis1+/- mice exhibit defects in various social behaviors.

Clearly, the authors put a lot of work into this study and some of the data is of very high quality. That said, the work is very descriptive, mainly characterizing cellular defects that result from altered Lis1 function, without key insights into the molecular mechanisms by which Lis1 is influencing cell morphogenesis. The characterization of actin dynamics and the possible suppression of defects with ROCK inhibitors comes closest to this, but does not provide major new mechanistic insights as to how Lis1 regulates RhoA/ROCK signaling. Finally, some of the data are not up to acceptable standards in the field. In light of these latter considerations, this paper is not acceptable for publication at EMBO Molecular Medicine. Without a major new insight into the molecular mechanisms by which Lis1 is functioning here, the work is really much better suited for a high-end anatomy journal, such as the Journal of Comparative Neurology.

Major Points
1. The FRAP curves look unusual, particularly for the Lis1+/- neurons treated with ROCK inhibitors. How many neurons were used in the FRAP experiments and how were regions of interest chosen for analysis? What statistical methods were used to compare the different treatment conditions?
2. How does Lis1 haploinsufficiency lead to elevated RhoA activity at the molecular level?

Resolving this seems to be a major issue in the field and one that would raise the profile of this work.
3. Several groups have shown that the phenotypes resulting from RhoA hyperactivation can be suppressed by infusing ROCK inhibitors either transcranially or intrathecally or by reducing gene dosage of ROCK2 (the major ROCK in the brain). Do these treatments affect the anatomical and behavioral changes observed in Lis1+/- mice?
4. Standard methods, including dendrite tracing and reconstruction and Sholl analysis must be performed to document the dendrite defects in the Lis1+/- neurons and the reported lack of defects in the conditional Lis1 neurons. These dendrite arbors are much more complex that cortical neurons and thus it is impossible to spot small but significant differences by eye.

Minor point
p 8 "Linear spine density in mutant neurons was reduced to 30% of control" should read "70% of control".

1st Revision - authors' response 20 December 2012

Referee #1 (General Remarks):

This is a well written paper and experimentally well-planned set of studies detailing a connection between Lis1 and regulation of spines in synaptogenesis and behavioural control in mice. The following are my comments on this paper:1-There are different descriptions of adolescent age in rodents which has implications on correct analysis of the data and its possible association with autism. Most rodent models of autism use P7-P14 as important times for appearance of autistic-like behaviour which correspond to ages 1-3 years in human brain development. Results presented here are reflective of adolescence(P20-P30) when most symptoms of schizophrenia occur i.e., 14-16 in male patients. Thus while the results obtained in these studies are novel they have more relevance to schizophrenia than autism ;2-are the dendritic spines described in page 5 similar to long immature dendrites observed in brains of subjects with fragile X syndrome ?

We thank the reviewer for the positive comments regarding our “well written paper and experimentally well-planned set of studies”.

The reviewer is quite correct in pointing out that most rodent models of autism attribute P7-P14 as the period of earliest onset of autistic-like behaviour. However, one cannot reliably test complex social interaction at these young ages, so that this conclusion regarding onset is instead based on ultrasonic vocalization data. All other autistic-like phenotypes are tested in mice at adolescent or even adult ages, and are nevertheless considered relevant to the autism spectrum (e.g., see Silverman, J. L., Yang, M., Lord, C. and Crawley, J. N. (2010) 'Behavioural phenotyping assays for mouse models of autism', *Nat Rev Neurosci* 11(7): 490-502). The three-chamber assay we use here—widely recognized as an autism-like paradigm--is typically performed starting at P21. The reviewer is correct that reduced socialization is also relevant to other neuropsychiatric disorders including schizophrenia and we so state in the paper. Although we point out that others have placed Lis1 in the molecular interaction pathways involving identified ASD genes, we do not claim that Lis1 deficiency is specific for ASD, only that Lis1 inactivation is a model for disorders in which socialization is prominently affected.

Referee #2 (Comments on Novelty/Model System):

The reason I gave the technical quality a medium rating is that it was difficult to assess the potential for error in many of the experiments. Some controls were not clearly described, and the details of many of the experimental procedures were fairly vague or scattered throughout the manuscript and difficult to follow. The experiments described are difficult and require substantial skill and an strong sense of the possible artifacts that might arise.

We appreciate the reviewer's general comments about Methods and Materials. In the previous submission, we tried to be succinct in writing Methods, citing where the detailed protocols may be found. We now realize that more detailed descriptions are needed and throughout the text we have included more details, some of which we specifically refer to in the remarks below. We hope that reviewer now finds our Methods to be more transparent.

Referee #2 (General Remarks):

The manuscript by Sudarov et al proposes that reduced Lis1 expression impacts dendritic protrusions (filopodia and spines). They make the argument that Lis1 loss results in less dynamic protrusions, and that this is linked to elevated RhoA activity.

The data in this paper represents a substantial effort, and these are complex and difficult experiments. The study presents potentially very important findings elucidating a role for Lis1 in the adolescent and adult brain (beyond its clear role in embryonic brain). In particular the behavioural defects are notable and interesting, and have implications for disorders like autism and schizophrenia.

We thank the reviewer for acknowledging our "substantial effort" and the "complex and difficult experiments". It is gratifying that the reviewer considers our newly discovered behavioural defects to be notable and interesting with implications for autism and schizophrenia.

The main concern with this manuscript is that it is sometimes difficult to determine exactly how experiments were done, and hard to gauge the potential for error in the analyses. Most of the confusion can be eliminated with better clarification/organization. I have indicated some ways that would make for an easier read and increase confidence in the findings.

We appreciate the comments regarding the need for greater clarity in the methodology used in these experiments. As noted above, both the text and methods sections have been amended to make the study easier to read.

Figure 1:
a) Readers are directed to an article that is not easily accessible for methods of the in situ imaging (Yang et al., 2010). More details should be provided in the methods section. Also, the methods for calculating filopodia motility were not easy to follow, and it was not clear if the same procedures were used in the brain and in cultures.

The Yang et al., 2010 study cited is published in *Nature Protocols*. The revision includes additional details in the Methods section. The methods for quantification were the same for both filopodia and spines *in vivo* and separate methods were used for quantification of filopodia motility *in vitro*.

b) In the introduction the authors indicate that while much is known about regulation of dendritic spine dynamics, less is known about regulation of dendritic filopodia. How are spines differentiated from filopodia in the experiments described here? Are all highly dynamic protrusions considered filopodia regardless of the age of the animals or cultures? What is the distinction between turnover (1C) and stability (1D-F)?

Filopodia are long and thin protrusions, whereas spines are shorter with clear enlargements termed the "head" of spines, or smaller stubby looking protrusions. Both spines and filopodia are considered highly dynamic. Turnover of filopodia and spines is a term used to describe how often a filopodium or spine is present and then lost during the time of imaging. Conversely, the stability is a term used to describe stable or constant presence of a protrusion (spine or filopodium) during the

time of imaging. These definitions are now included in the Methods section of the revised manuscript.

c) The time lapse intervals, duration of imaging, and time between imaging sessions for the four graphs in this figure need to be more clearly defined, either in the text, the legends, or the methods. It takes a lot of effort on the part of the reader to try to elucidate the relevant specifics for each set of data.

We appreciate the comment and have made this more clear in the figure, methods and legend.

d) Were there fewer filopodia/protrusions in general, as was observed in hippocampal cultures (Fig 2)?

Yes, there were fewer filopodia in P21 barrel cortex. We thank the reviewer for pointing this out. A graph that depicts quantification of filopodia density is now included in Sup. Fig 1B.

Figure 2:
a) The cells in A and B look like different types of cells. B looks more like an immature neuron, a glial cell, or some other type of neuron rather than a hippocampal pyramidal neuron. (see Benson et al, J. Neurocytology, 1994 May;23(5):279-95).

The previous panel focused on dendrites and did not always show the entire neuron so that filopodia could be better appreciated. In the revised figure, we have replaced those panels with TuJ1/GFP double immunocytochemistry to demonstrate that cells analysed are indeed immature hippocampal pyramidal neurons.

b) Where along the dendritic shaft were time-lapse sequences obtained? Were the regions randomly selected? What length of dendritic shaft was used for these studies? Are there differences in the density of protrusions/length? If dendrites are longer, there could be equal numbers of protrusions overall, but more sparsely positioned. This would still be interesting but may reflect an alteration in dendritic growth rather than spine dynamics.

Time lapse sequences were obtained ~50μm from the cell body. The neurons from which the regions were chosen were randomly selected. At the time points in which the present measurements were made, there were no differences in dendrite length between genotypes. If anything, *Lis1+/-* dendrites would be slightly shorter and by the reviewer's suggested scenario protrusions should be more dense than wild type, not less. As we previously showed, after 24 hours in culture, *Lis1+/-* neurites were shorter than wild type by 15% and were no different thereafter (Kholmanskikh et al., 2003).

c) The legend for D indicates the data are from both 2DIV and 10DIV, but the graph only shows two bars (Lis1+/+ and Lis1+/-).

We apologize for this oversight, the legend has been fixed to state only DIV2 quantification.

d) There is confusing reasoning in the text describing the RhoA experiments: "Because Lis1 haploinsufficiency leads to elevated RhoA activity, and constitutive activation of RhoA negatively regulates spine formation and stability, we asked whether up regulated RhoA is responsible for diminished filopodial motility in Lis1+/- neurons". If overactive RhoA prevents spine formation and destabilizes spines, why would the same event reduce motility? This needs to be clarified, or the relationship between motility and stability needs to be clarified.

We thank the reviewer for pointing this out. The constitutive activation experiments referred to in the text involved the transfer of a constitutively active, mutant form of RhoA into primary neurons.

This is a molecular manipulation that does not always recapitulate all aspects of naturally occurring RhoA pathway regulation. Thus, we chose to examine the involvement of this small GTPase because we knew RhoA activity to be elevated in Lis1 haploinsufficiency and because RhoA constitutive activation had been shown to have an impact on spine formation and dynamics, but did not have a preconceived notion of what the Lis1 involvement would be. We have re-worded this statement to avoid confusion.

e) It seems odd that ROCK inhibitors would have no impact on filopodia in control neurons. Is RhoA not involved in preventing filopodial motility under normal circumstances?

Indeed, the present and previous studies (Nakayama et al., 2000; Govek et al., 2004) have suggested that inhibition of the RhoA pathway in wild type neurons does not affect protrusion density and length (including both filopodia and spines). We and others hypothesize that under normal conditions, the RhoA pathway achieves a steady state in maintaining dendritic morphology including spines and that inhibiting the ROCK downstream effector does not prevent compensatory adjustments to the perturbation. In contrast, when the GTPase signalling pathway is in a disregulated state, inhibition of basally elevated RhoA activity is seen to restore these actin-rich structures.

Figure 3:
a) Are there error bars for the data in 3D?
b) Is the data statistically significant?
c) How does actin polymerization rate contribute to actin turnover?
d) Was turnover measured in spines or filopodia?

We appreciate the helpful comments regarding Figure 3. The following changes have been made in the revised manuscript:

a) The error bars for the data in 3D have been made more visible
b) The data are statistically significant and that is now clearly stated in the text
c) Actin turnover is a dynamic process that is dependent on actin polymerization, depolymerization and capping. Therefore, if any of those processes are abnormal it will negatively affect actin turnover. In order to be more precise we amended text to state that we found defects in actin polymerization and avoid the more global term "turnover".
d) Actin polymerization was measured in both spines and filopodia, however the majority of quantified protrusions (~80%) are spines

Figure 4:
E and J should be labelled CA1 and barrel cortex to avoid confusion.

Thank you for the suggestion. It is done.

Figure 5:
a) Are the images confocal images? If not which of the Z-steps was used for analysis of overlap?
b) It would possibly be more informative if the numbers of vGlut1+/PSD95- and vGlut1-/PSD95+ puncta were included in the analysis.
c) Defects in localization of PSD95 or vGlut1 rather than altered spine behaviour could explain this result.

These images were collected using a spinning-disc confocal microscope. We collected 0.5µm Z steps and then analysed maximum projections. Our primary goal was to address the number of potential functional synapses that are forming and are defined as the clusters where there is clear vGlut1/PSD95 overlap. We didn't discriminate whether the puncta were found on dendritic shafts or spines and therefore changes in PDS95 or vGlut1 localization does not account for the observed lag in synaptic cluster formation.

We have looked at the localization of synaptic clusters and found that a greater proportion of

clusters are found on dendrite shafts in the Lis1 mutant neurons. However, this information will not distinguish whether the mislocalization arises because there was no filopodium or spine to begin with or because transport into the protrusion was faulty. We therefore have not included these data in the manuscript, but can add it to supplementary data if requested.

Figure 6:
a) How do the mice spend time "in close interaction" with an empty cup? This is probably a simple thing, but could use more explanation.
b) If possible, it would be interesting to inhibit RhoA to determine if the behaviours are rescued.

Thank you for pointing this out. We included more details in the methods section. When we say "in close interaction" we mean that the subject mouse was physically interacting with the wire cup whether it was empty or with another mouse in it.

We agree with the reviewer that it would be interesting to investigate the impact of RhoA inhibition at the systems level. Therefore, in the revised manuscript, we have used systemic administration of ROCK inhibitor compared to saline control to show that dendritic spines are indeed restored 24 hours after a single I.P. injection of the inhibitor. To pursue behavioural outcomes after ROCK inhibition would require extensive investigation and validation of the drug delivery mode and dosing schedule, and control for the somatic vs. central nervous system effects on behaviour that is beyond the scope of this manuscript.

Figure 7:
a) There should be some way of determining whether Lis1 mRNA or protein levels were in fact reduced in the intact hippocampus and in the cultured neurons. This might require is situ hybridization or immunostaining. Western or northern blots might suffice for cultures.

We thank the reviewer for suggesting we provide this supporting data. Sup Fig 5A is added, which shows Western blots of dissociated hippocampal Lis1 in $Lis1^{fl/fl}$ cell cultures that were treated with cre lentivirus or inactive cre lentivirus to demonstrate Lis1 loss. In addition, brain lysates were collected from $Lis1^{fl/+}$ and *Lis1cko* P30 animals. We carefully dissected CA1 regions and used their lysates for Western blots shown in Sup Fig 5B. We performed anti-Lis1 immunohistochemistry on free floating sections of $Lis1^{fl/+}$ and *Lis1cko* P30 animals to demonstrate reduced levels of Lis1 in Sup Fig 5C-F.

Discussion:

Confusing concept again:
"...we found a significant decrease in spine density in adolescent mutant animals, together with a reduced rate of turnover. Therefor, Lis1 haploinsufficient neurons fail to undergo adolescent pruning of dendritic spines and continue to steadily accumulate spines throughout adolescence, finally by adulthood reaching the same density as wild type neurons."

If there is no increase in the formation of spines, but there is reduced elimination as suggested by Figure 1, why would spine density be lower in mutant adolescent animals? Wouldn't the density increase, because spines are not being eliminated as efficiently? The fact that there is a decreased spine density suggests that there is a defect in spine formation or maintenance.

We appreciate the reviewer's point. Indeed, we infer that filopodia and spine formation rates must be lower than wild type (the data already show prolonged maintenance), as indicated by the lag in synaptic cluster formation in *Lis1+/-* neuronal cultures and reduced actin polymerization rates in spines as indicated by FRAP. However, over the time periods that it is technically feasible to observe filopodia and spine motility, we see only trends toward reduced formation, while reductions in elimination rates are statistically significant. On balance, these effects are such that dendrite spine

densities "catch up" to wild type levels in the adult *Lis1+/-* neurons. If formation rate reductions were more robust, we would not see this catch-up phenomenon.

Referee #3 (Comments on Novelty/Model System):

The work is very descriptive and does not provide important new mechanistic insights into how reduced function of Lis1 controls dendritic spine and dendrite formation.

My recommendation below is really at the bottom end of the enthusiasm range. It would need very significant additional work to reach the profile one would expect for EMBO Mol Med.

We hope that the clarifications and additional data provided in the revised manuscript will improve the reviewer's opinion.

Referee #3 (General Remarks):

In this manuscript, Sudarov et al. characterize the cellular and anatomical defects that result from loss or reduced function of the Lis1 protein. Transcranial imaging is used to shown that dendritic filopodia in apical dendrites of layer V barrel cortex neurons are much less dynamic in formation and turnover in Lis1+/- animals during both adolescence and in early adulthood. Moreover, the extension and retraction of individual filopodia is greatly reduced in Lis1+/- neurons relative to wt controls. The author demonstrate that inhibition of ROCK (and not RhoA as stated in the section title) increases filopodial motility. ROCK inhibitors also appears to modestly rescue defects in actin dynamics, although it is hard to gauge the magnitude of this effect based on the spare experimental details given. Lis1+/- mice also have reduced dendritic spine density. The claim is made that hippocampal dendrite arbors are altered in the Lis1+/- mice, but the descriptions are not backed up with rigorous measurements. Similarly, the claim is made that conditional knockout of Lis1 in adulthood does not affect dendrite structure, but this is not backed up with data. The authors go on to show that the Lis1+/- mice exhibit defects in various social behaviours.

Clearly, the authors put a lot of work into this study and some of the data is of very high quality. That said, the work is very descriptive, mainly characterizing cellular defects that result from altered Lis1 function, without key insights into the molecular mechanisms by which Lis1 is influencing cell morphogenesis. The characterization of actin dynamics and the possible suppression of defects with ROCK inhibitors comes closest to this, but does not provide major new mechanistic insights as to how Lis1 regulates RhoA/ROCK signalling. Finally, some of the data are not up to acceptable standards in the field. In light of these latter considerations, this paper is not acceptable for publication at EMBO Molecular Medicine. Without a major new insight into the molecular mechanisms by which Lis1 is functioning here, the work is really much better suited for a high-end anatomy journal, such as the Journal of Comparative Neurology.

We appreciate the reviewer recognizes that we "put a lot of work into this study" and that data "of very high quality" are included.

Major Points

1. The FRAP curves look unusual, particularly for the Lis1+/- neurons treated with ROCK inhibitors. How many neurons were used in the FRAP experiments and how were regions of interest chosen for analysis? What statistical methods were used to compare the different treatment conditions?

We are not entirely certain what the reviewer found unusual in the FRAP curves. We have consulted a number of references in which FRAP data are presented and the representation is similar to ours

[Misteli et al., 2000 (Nature); Campbell and Knight, 2007 (Microscopy Research and Technique); Tatavarty et al., 2009 (Plos One)]. The number of total protrusions used to generate each curve is indicated in the text. The regions of interest were chosen randomly but always within 100-200μm of the cell body of DIV14 dissociated hippocampal pyramidal neurons. We used a Standard *t*-test to compare different treatment conditions.

2. How does Lis1 haploinsufficiency lead to elevated RhoA activity at the molecular level? Resolving this seems to be a major issue in the field and one that would raise the profile of this work.

We agree with the reviewer that this is a crucial issue to address and others in our lab are endeavouring to do so. While we believe we have made significant progress in this area, we know that answering this important question regarding the molecular mechanism of RhoA GTPase disregulation is a complex study by itself and is well beyond the scope of the present paper. Nevertheless, we submit that the current work identifies for the first time the developmental impact of Lis1, and its nature, on synaptogenesis and the dynamics of actin-rich structures in post-migrational neurons. It provides the first insights into how Lis1 function may intersect with genes implicated in cognitive disorders affecting social interaction. The work is unusual in that it bridges investigation from the molecular genetic, to dynamic cellular *in vitro* and *in vivo* systems levels, ultimately to behavioural outcome. This approach is well beyond the depth of the purely anatomical/histological investigations appearing in *J. Comp. Neuro.*

3. Several groups have shown that the phenotypes resulting from RhoA hyper activation can be suppressed by infusing ROCK inhibitors either transcranially or intrathecally or by reducing gene dosage of ROCK2 (the major ROCK in the brain). Do these treatments affect the anatomical and behavioural changes observed in Lis1+/- mice?

We thank the reviewer for this excellent suggestion. In the revised manuscript, we treated adolescent (P20) *Lis1+/+* and *Lis1+/-* animals with ROCK inhibitor Y-27632 and 24 hrs later collected the tissue for Golgi staining to analyse anatomical changes in CA1 pyramidal neurons. The data are added in Fig 4 and text. As discussed above, extension of this anatomical observation to behavioural experiments, if possible at all, would require generation and extensive validation of a complex paradigm that is beyond the scope of this paper.

4. Standard methods, including dendrite tracing and reconstruction and Sholl analysis must be performed to document the dendrite defects in the Lis1+/- neurons and the reported lack of defects in the conditional Lis1 neurons. These dendrite arbors are much more complex that cortical neurons and thus it is impossible to spot small but significant differences by eye.

We thank the reviewer for this suggestion and have now completed Sholl analysis for *Lis1+/+*, *Lis1+/-*, *Lis1*$^{fl/+}$ and *Lis1cko* animals and included the results in Fig 4 and Fig 7.

Minor point

p 8 "Linear spine density in mutant neurons was reduced to 30% of control" should read "70% of control".

Thank you for pointing this out. It is now amended in the text.

Again, the authors thank the reviewers for their thoughtful comments that have prompted gathering additional data and further clarification of points to significantly strengthen the manuscript. We hope that you will find the paper now suitable for publication in *EMBO Molecular Medicine*.

3rd Editorial Decision 20 January 2013

We have now received the comments from the three Reviewers whom we asked to evaluate your revised manuscript.

You will see that while Reviewer is now satisfied with your revision, Reviewers 2 and 3 still have significant concerns that prevent us from considering publication at this time.

Both are unsatisfied with the level of mechanistic insight into how Lis1 function controls dendritic spine and dendrite formation. While Reviewer 2 is open to a new revision, Reviewer 3 does not support publication of your work.

We feel that the detailed mechanistic elucidation of how Lis1 regulates dendritic protrusions is not required for publication of your work given its pathophysiological implications. It is of the essence, however, that you improve the manuscript along the lines suggested by Reviewer 2 by providing a solid discussion and possibly a schematic. I would also encourage you to include further data, if available, to improve this aspect.

Reviewer 2 also feels that the distinction between spines and filopodia remains confusing and that these structures should be clearly identified in the images; s/he asks whether the loss of Lis1 changes the spine/filopodia ratio. Finally Reviewer 2 finds that the measurement of turnover remains unclear and requires clarification in that respect.

As you know, we would normally not allow a second revision. I am prepared, however, to give you the opportunity to improve your manuscript further, with the understanding that Reviewer 2's concerns must be fully and carefully addressed. This, of course, will possibly entail a further round of review with Reviewer 2.

I look forward to seeing a revised form of your manuscript as soon as possible.

***** Reviewer's comments *****

Referee #2 (General Remarks):

The authors have addressed most of my concerns, however the distinction between spines and filopodia remains confusing. Spines and filopodia should be identified in the images throughout. Does Lis1 loss change the spine/filopodia ratio?

Also, the measurement of turnover remains unclear. Turnover apparently measures "how often a protrusion is present then lost during imaging", but the data in Figure 1C are presented as a percentage, similar to formation and elimination. Is turnover simply the sum of the percentage of all spines that are formed OR eliminated during imaging? Or is it a measure of the number of times a protrusion forms and is eliminated?

Finally, the hypothesis for how Lis1 is regulating dendritic protrusions is vague. The paper would benefit from a cartoon to clarify how RhoA and Lis1 pathways are thought to interact to regulate protrusion, motility, elimination, stability.

Referee #3 (General Remarks):

My previous criticism is that this work is very descriptive and does not provide important new mechanistic insights as to how Lis1 function controls dendritic spine and dendrite formation. The authors have attempted to address some issues, but the major issue of Lis1 regulates RhoA/Rock signaling is not addressed.

© EMBO

In addition, the authors provide new evidence that dendritic arbors are significantly reduced in Lis1+/- animals but largely unaffected in Lis1cko animals. It is important to note that the loss of Lis1 protein from Lis1cko animals was not confirmed by immunoblotting.

Overall, there is a lot of data presented and some of it is of high quality. That said,the work is very descriptive and does not lead to major new mechanistic insights regarding the mechanism of spine and dendrite formation by Lis1. As such it does not reach the standard one might expect of EMBO Molecular Medicine.

2nd Revision - authors' response 29 January 2013

Referee #1 (General Remarks):

We thank the reviewer for his/her positive remarks regarding our manuscript.

Referee #2 (General Remarks):

The authors have addressed most of my concerns, however the distinction between spines and filopodia remains confusing. Spines and filopodia should be identified in the images throughout. Does Lis1 loss change the spine/filopodia ratio?

We have now labelled additional filopodia and spines in Figure 1 and Sup. Fig. 1 to illustrate the difference in morphology. Also, we have added the quantification of spine/filopodia ratios at P21 and P30 stages to Sup. Fig. 1. We did not find any differences in the filopodia/spine ratio between *Lis1+/+* and *Lis1+/-* mice at P21 or P30 stages. Therefore reduction in filopodia associated with Lis1 happloinsufficiency is accompanied by a proportional reduction in dendritic spines.

Also, the measurement of turnover remains unclear. Turnover apparently measures "how often a protrusion is present then lost during imaging", but the data in Figure 1C are presented as a percentage, similar to formation and elimination. Is turnover simply the sum of the percentage of all spines that are formed OR eliminated during imaging? Or is it a measure of the number of times a protrusion forms and is eliminated?

We apologize that in our initial revision we did not sufficiently explain our use of the term “turnover” rate. As the reviewer pointed out correctly, the turnover in Fig. 1C represents the sum of the percentage of all filopodia that are formed and eliminated during imaging. It was calculated as $(N_{gained} + N_{lost})/(2 \times N_{total})$. This term was also used in other studies (such as: *Transient and persistent dendritic spines in the neocortex* in vivo. Holtmaat AJ, Trachtenberg JT, Wilbrecht L, Shepherd GM, Zhang X, Knott GW, Svoboda K. Neuron. 2005 Jan 20;45:279-91.; and *Long-term in vivo imaging of experience-dependent synaptic plasticity in adult cortex*. Trachtenberg JT, Chen BE, Knott GW, Feng G, Sanes JR, Welker E, Svoboda K. Nature. 2002 Dec 19-26;420:788-94.) Similar to our manuscript, the authors in these studies used the term “Turnover ratios” to represent the fraction of filopodia or spines appearing and disappearing over days. We have now added this explanation to methods section. It is our hope that reviewer agrees with us that turnover rate is an important measurement as it directly addresses the plasticity of dendritic protrusions.

Finally, the hypothesis for how Lis1 is regulating dendritic protrusions is vague. The paper would benefit from a cartoon to clarify how RhoA and Lis1 pathways are thought to interact to regulate protrusion, motility, elimination, stability.

We thank the reviewer for the suggestion to improve our manuscript by providing a cartoon. It is our hope that our new schematic figure aids in clarification of how RhoA and Lis1 pathways regulate plasticity of dendritic protrusions.

Referee #3 (General Remarks):

My previous criticism is that this work is very descriptive and does not provide important new mechanistic insights as to how Lis1 function controls dendritic spine and dendrite formation. The authors have attempted to address some issues, but the major issue of Lis1 regulates RhoA/Rock signalling is not addressed.

We respectfully disagree with the Reviewer 3 statement that our work does not provide important new mechanistic insights as to how Lis1 functions to control plasticity of dendritic protrusions. This is the first demonstration that loss of Lis1 function results in altered dendritic protrusion dynamics and behavioural deficits via disregulated RhoA activity. Specifically, using *Lis1* heterozygous and adolescent *Lis1* conditional knock out mutants we find distinct deficits in dendritic protrusion plasticity that are related to synapse formation and, on a systems level, affect social behaviour, even when the Lis1 deficit is induced after neuronal migration is complete. Using both *in vitro* and sophisticated *in vivo* imaging tools we are able to show that *Lis1* haploinsufficiency results in reduced motility of dendritic filopodia and in the altered pace of dendritic spine formation/elimination. Using FRAP methods we demonstrate that actin polymerization, which is necessary for proper motility of protrusions, is indeed disturbed in *Lis1*+/– neurons. Using pharmacological reagents we show that elevated RhoA activity is largely responsible for decreased filopodial motility as well as defects in actin polymerization. Moreover, systemic administration of a Rock inhibitor can restore spine density in the *Lis1+/–* juvenile brain. Finally, by utilizing behaviour tests for mice we find deficits in social interactions in our mutants, including when *Lis1* is conditionally inactivated postnatally. We agree with the reviewer that how Lis1 regulates RhoA/Rock signalling is a crucial issue. We believe that addressing these issues requires detailed experiments in the next phase of these investigations and is beyond the scope of the present manuscript.

In addition, the authors provide new evidence that dendritic arbors are significantly reduced in Lis1+/- animals but largely unaffected in Lis1cko animals. It is important to note that the loss of Lis1 protein from Lis1cko animals was not confirmed by immunoblotting.

We would like to point out that Supplemental Figure 5 submitted with revised manuscript demonstrated the loss of Lis1 protein in *Lis1* conditional knock out neurons, both in dissociated hippocampal cultures by Western blot and in the CA1 region of the *CamKII-Cre;Lis1cko* hippocampus using both Western blot and immunohistochemistry.

Overall, there is a lot of data presented and some of it is of high quality. That said, the work is very descriptive and does not lead to major new mechanistic insights regarding the mechanism of spine and dendrite formation by Lis1. As such it does not reach the standard one might expect of EMBO Molecular Medicine.

We appreciate that reviewer feels that we presented a lot of data and of high quality.

Again, we thank the Editors for the opportunity to submit this second revision of the paper. We also thank the Reviewers for their thoughtful comments that have led to significant strengthening of the manuscript. We hope that with this further clarification, new summary figure and new data, you will find the paper now suitable for publication in *EMBO Molecular Medicine*.

© EMBO

Accept 31 January 2013

The Reviewer returned his/her evaluation on your revised manuscript virtually overnight. I am thus pleased to inform you that your manuscript is accepted for publication and is now being sent to our publisher to be included in the next available issue of EMBO Molecular Medicine. It has been a long journey but with a happy ending!

Congratulations on your interesting work,

***** Reviewer's comments *****

Referee #2 (General Remarks):

This will be of interest to a wide range of readers. It is not clear how defective filopodial dynamics in adolescence produces altered social behavior in adults without apparent reduction or increased spine density, however, this is a good start to the characterization of a potentially important molecular link between Lis1 and the development of autism and/or schizophrenia.
