# Supplementary material for: Lis1 controls dynamics of neuronal filopodia and spines to impact synaptogenesis and social behaviour
Source: EMBO Mol Med. 2013 Mar 11;5(4):591–607. doi: 10.1002/emmm.201202106 (PMC3628102; doi:10.1002/emmm.201202106)
Supplement: Supplementary file 2 [file emmm0005-0591-sd2.pdf]

**TABLE OF CONTENTS**

**Sup. Fig. 1** Quantification of filopodia dynamics and density using images collected with two photon *in vivo* imaging at P21. *In vivo* two photon imaging of dendritic spines in *Lis1*+/+ and *Lis1*+/– P30 mice. Quantification of filopodia/spine ratio at P21 and P30.

**Sup. Fig. 2** (A, B) Protrusion tips are marked in each frame by a cross. Motile protrusions are represented by wide distribution of crosses in Lis1+/+ neurons (A), in contrast less motile protrusions in *Lis1*+/– neurons have crosses clustered together (B).

**Sup. Fig. 3** Spine density in mature dissociated hippocampal neurons at 21 days *in vitro* appears similar between *Lis1*+/+ and *Lis1*+/– animals.

**Sup. Fig. 4** Gross morphology as evidenced by NeuN immunostaining of hippocampus appears similar in $Lis1^{flox/+}$ and *Lis1cko* P28 animals.

**Sup. Fig. 5** Western blot analysis of loss of Lis1 in the hippocampal cultured neurons and CA1 dissected hippocampal lysates at P28. IHC for anti-Lis1.

**Sup. Video 1**. Time-lapse movies of *Lis1*+/+ and *Lis1*+/– immature dissociated hippocampal cultures at 2 days *in vitro*.

**Sup. Video 2**. Time-lapse movies of *Lis1*+/– DIV2 dissociated hippocampal neuron before and after 1 hour incubation with ROCK inhibitors.

**Sup. Video 3.** FRAP time-lapse movies of *Lis1*+/+, *Lis1*+/– before and after 1 hour incubation with ROCK inhibitors of DIV12 dissociated hippocampal neurons infected with AAV2-GFP-Actin monomer.

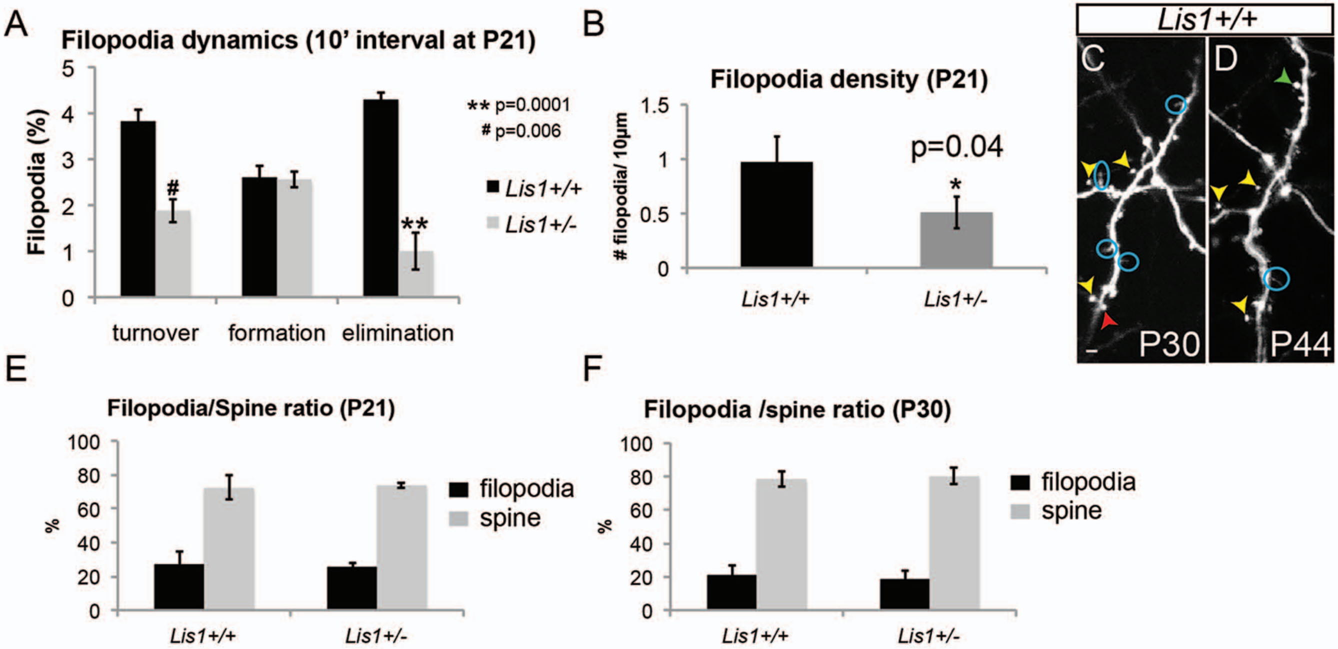


Sudarov et al., Suppl. Fig. 1

Sup. Fig. 1 (A) Quantification of filopodia dynamics using two photon in vivo imaging at P21. Filopodia elimination (P=0.0001) and turnover (P=0.006) rates over 10 mins are lower in Lis1+/– mice. Formation rates were similar (P=0.95). (B) Quantification of filopodia density using images collected with two photon in vivo imaging at P21 (n=3 animals/genotype; P=0.04). (C, D) In vivo two photon imaging of dendritic spines in Lis1+/+ and Lis1+/– P30 mice. Same area was imaged at P30 and P44. Yellow, green, and red arrowheads indicate stable, formed and eliminated spines, respectively. For comparison, blue circles indicate dendritic filopodia. Scale bar: 1 µm. (E, F) Filopodia/spine ratio at P21 and P30 ages are compared between Lis1+/+ and +/- genotypes (n=3 animals/genotype).

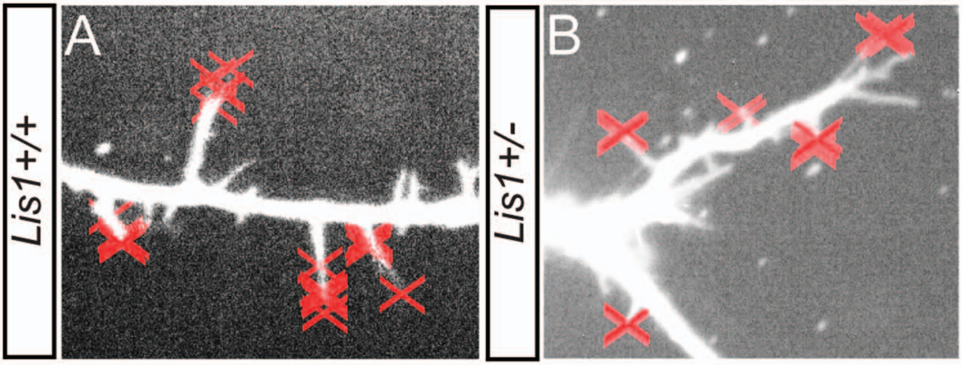


Sudarov et al., Suppl. Fig 2

Sup. Fig. 2 (A, B) Protrusion tips are marked in each frame by a cross. Motile protrusions are represented by wide distribution of crosses in Lis1+/+ neurons (A), in contrast less motile protrusions in Lis1+/– neurons have crosses clustered together

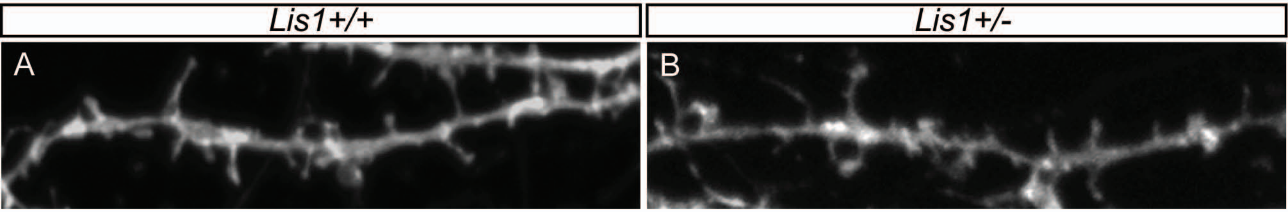


Sudarov et al. Suppl. Fig. 3

Sup. Fig. 3 (A, B) Spine density in mature dissociated hippocampal neurons at 21 days in vitro appears similar between Lis1+/+ and Lis1+/– animals.

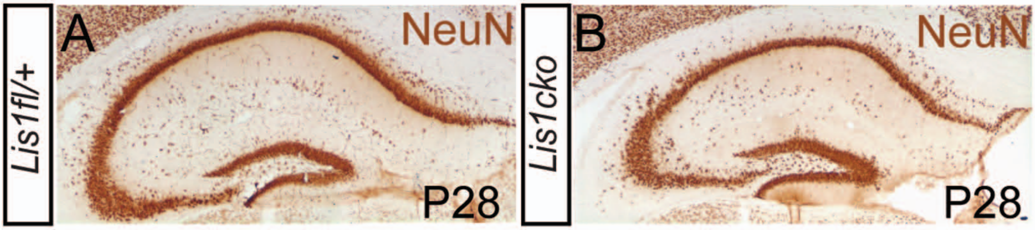


Sudarov et al., Suppl. Fig. 4

Sup. Fig. 4 (A, B) Gross morphology as evidenced by NeuN immunostaining of hippocampus appears similar in Lis1flox/+ and Lis1cko P28 animals.

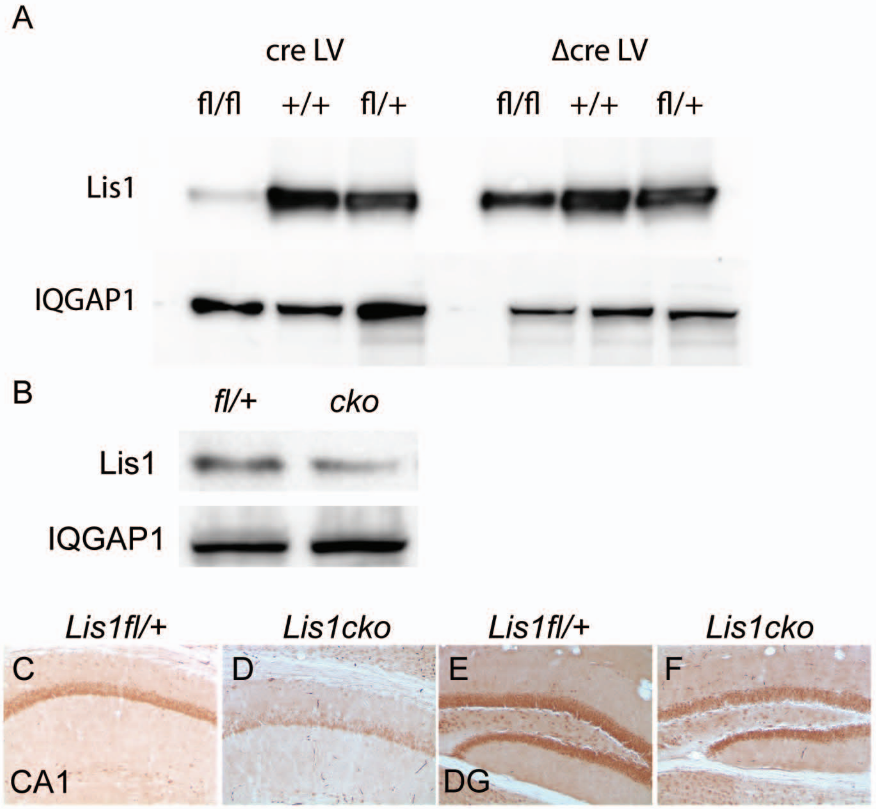


Sudarov et al. Suppl. Fig. 5

Sup. Fig. 5 Western blot analysis of loss of Lis1 in the hippocampal cultured neurons that were infected with Cre or ΔCre lentivirus (A) and from CA1 dissected hippocampal lysates at P28 (B). IHC for anti-Lis1 shows that in Lis1cko mice (D), Lis1 expression is reduced in the CA1 region when compared to Lis1flox/+ (C) and not in the dentate gyrus (DG) of the hippocampus (E,F).
